# PLD-dependent phosphatidic acid microdomains are signaling platforms for podosome formation

**DOI:** 10.1038/s41598-019-39358-0

**Published:** 2019-03-05

**Authors:** Matteo Bolomini-Vittori, Svenja F. B. Mennens, Ben Joosten, Jack Fransen, Guangwei Du, Koen van den Dries, Alessandra Cambi

**Affiliations:** 1 Department of Cell Biology, Radboud Institute for Molecular Life Sciences, Radboud University Medical Center, Nijmegen, The Netherlands; 2 Microscopic Imaging Center, Radboud Institute for Molecular Life Sciences, Radboud University Medical Center, Nijmegen, The Netherlands; 3 Department of Integrative Biology and Pharmacology, University of Texas Health Science Center, Houston, Texas USA

## Abstract

Local membrane phospholipid enrichment serves as docking platform for signaling proteins involved in many processes including cell adhesion and migration. Tissue-resident dendritic cells (DCs) assemble actomyosin-based structures called podosomes, which mediate adhesion and degradation of extracellular matrix for migration and antigen sampling. Recent evidence suggested the involvement of phospholipase D (PLD) and its product phosphatidic acid (PA) in podosome formation, but the spatiotemporal control of this process is poorly characterized. Here we determined the role of PLD1 and PLD2 isoforms in regulating podosome formation and dynamics in human primary DCs by combining PLD pharmacological inhibition with a fluorescent PA sensor and fluorescence microscopy. We found that ongoing PLD2 activity is required for the maintenance of podosomes, whereas both PLD1 and PLD2 control the early stages of podosome assembly. Furthermore, we captured the formation of PA microdomains accumulating at the membrane cytoplasmic leaflet of living DCs, in dynamic coordination with nascent podosome actin cores. Finally, we show that both PLD1 and PLD2 activity are important for podosome-mediated matrix degradation. Our results provide novel insight into the isoform-specific spatiotemporal regulation of PLD activity and further our understanding of the role of cell membrane phospholipids in controlling localized actin polymerization and cell protrusion.

## Introduction

Actomyosin-mediated reorganization of the cell cytoskeleton is essential for cell migration and invasion. Podosomes are the most prominent actomyosin structures in myeloid cells such as osteoclasts, immature dendritic cells (DCs) and macrophages^1–3^. In addition, they have been described in Src-transformed fibroblasts^4,5^, smooth muscle cells^6^ endothelial cells^7^ and megakaryocytes^8,9^. DCs, as orchestrators of both innate and adaptive immune responses, make podosomes to breach basal membranes and sample peripheral tissues for invading pathogens^10^. Upon encountering an antigen, immature DCs become activated to turn into mature DCs, which quickly disassemble podosomes and migrate to a regional lymph node, where they present the antigen to T cells, thereby initiating an immune response^11^. Structurally, podosomes present several analogies with invadopodia, which are actomyosin protrusions that facilitate cancer cell invasion^12,13^, emphasizing the pathophysiological relevance of these cytoskeletal structures.

Podosomes are multimolecular mechanosensory structures with a complex architecture consisting of a protrusive actin-rich core that displays radial actomyosin connections to neighboring podosomes or to the membrane^14^. Each podosome core is surrounded by regulatory proteins, adaptor molecules and integrins forming the so-called podosome ring, which connects these cytoskeletal structures to the extracellular matrix^14,15^. Podosomes are formed in response to a plethora of extracellular signals that converge to intracellular molecules such as protein kinase C (PKC), guanine nucleotide exchange factors, Src, Arf and Rho family members. These molecules induce recruitment of effector proteins including core components of podosomes, such as WASP and Arp2/3, or ring components of podosomes, such as talin, vinculin and myosin IIa^16–18^. How these input signals are integrated and regulated to control podosome formation and spatiotemporal organization remains poorly described.

Phospholipase D (PLD) is a phosphodiesterase that catalyzes the transphosphatidylation of phosphatidylcholine (PC) to phosphatidic acid (PA) and choline. The PLD family consists of six members of which PLD1 and PLD2 are the most abundant and the only ones with established catalytic activity^19,20^. PLD1, PLD2, and their product PA, are involved in a variety of cellular processes including vesicular trafficking, actin rearrangement, cell proliferation, differentiation, and migration, in both physiological and pathological conditions^21,22^. As effector of RhoA, Rac1 and Cdc42, PLD1 has been shown to play a role in both leukocyte adhesion and migration^23–25^. Interestingly, PLD2 is involved in leukocyte migration with functions similar to PLD1, but its activity does not depend on RhoA^26^. Recently, PLD activity has been reported to control podosome formation in mouse megakaryocytes, in which PLD1 KO, PLD2 KO, and double knockdown resulted in reduced actin filaments and reduced number of podosomes^27^. To date, however, a role for PLD1 and PLD2 in controlling podosome formation in human DCs has not been demonstrated. Moreover, although a differential spatiotemporal control of cell adhesion by PLD isoforms has been proposed^24,28^, the specific involvement of PLD1 and PLD2 isoforms in the control of podosome formation and podosome-driven matrix degradation is still unknown.

Phospholipids are essential membrane components not only for their intrinsic structural role, but also for their important role as second messengers. In eukaryotic cells, PA is a lipid messenger that has been found to change membrane curvature and to modulate the activity of different molecules, including vinculin, Arp2/3 and phosphatidylinositol 4-phosphate-5 kinase (PI4P5K)^29–32^. Membrane phospholipids have been demonstrated to organize in microdomains and to work as signaling platforms for different processes such as vesicular trafficking or autophagy^33–38^. PA confinement in microdomains during vesicular fusion to the plasma membrane has been demonstrated during exocytosis process^39,40^. Still, although PA plays a role in many cellular processes, its direct visualization and involvement at the site of podosome formation as well as the existence of PA microdomains at the site of podosomes have never been demonstrated.

In this study we sought to determine the specific role of PLD1 and PLD2 and their product PA in regulating podosome formation and dynamics in human primary DCs by combining PLD biochemical inhibition with a fluorescent PA sensor with improved sensitivity^41^ and fluorescence microscopy. Our results demonstrate that of the two PLD isoforms only PLD2 controls podosome maintenance at steady state, whereas both PLD1 and PLD2 are involved in controlling the early stages of podosome assembly induced by adhesion, microtubule reformation or chemoattractant. Furthermore, we captured the formation of PA microdomains accumulating at the membrane cytoplasmic leaflet of living DCs, in dynamic coordination with nascent podosome actin cores. Finally, we show that both PLD1 and PLD2 activity is important for podosome-dependent matrix degradation. Our results provide novel relevant information to further our understanding of the role of cell membrane phospholipids in controlling localized podosome formation.

## Results

### PA production is essential for podosome maintenance in resting DCs

**Figure 1:**
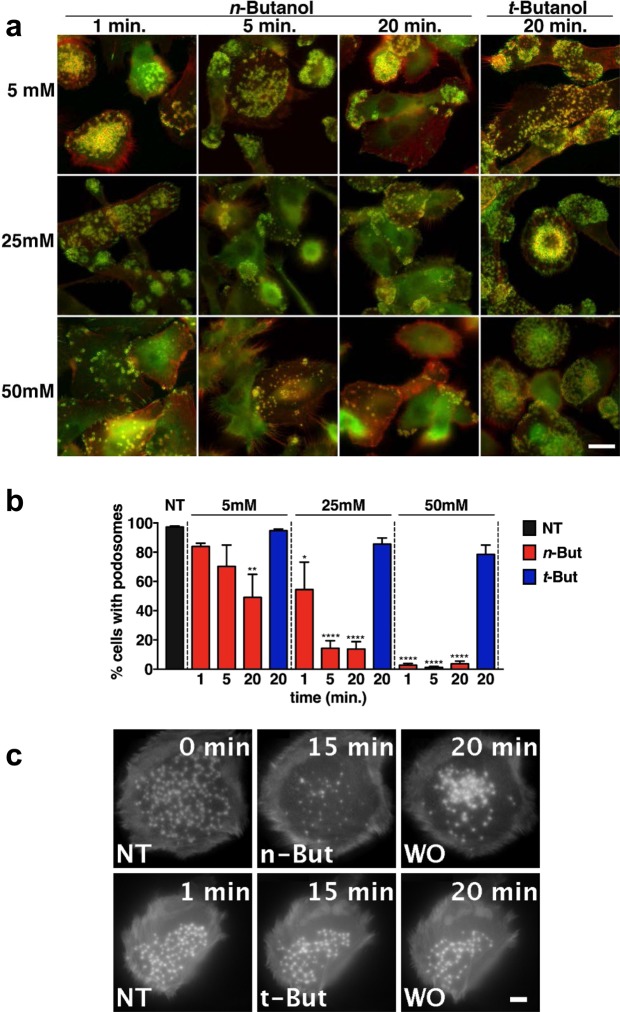
PA production is essential for podosome maintenance in resting DCs. (**a**) Representative widefield images of DCs treated for indicated times and at indicated doses with *n*-butanol or *t*-butanol. Vinculin is shown in green and F-actin in red. Scale bar represents 20 µm. (Bb) Percentage of cells with podosomes in DCs treated for indicated times and at indicated doses with *n*-butanol or *t*-butanol. Three samples of each experimental condition were prepared for each experiment. A minimum of 25 cells were imaged and analyzed for each sample. Three independent experiments were performed. Bars represent mean with SEM. Statistical significance was tested with one-way ANOVA with post-hoc Bonferroni’s multiple comparisons test. Asterisks indicate statistical significant differences compared to non-treated control (NT). Adjusted P-values: *0.0172, **0.0055, ****0.0001. (**c**) Representative confocal images of live cell imaging of DCs transfected with LifeAct-GFP. After 10 minutes of imaging, DCs were treated with 25 mM *n*-butanol (upper row) or 25 mM *t*-butanol (lower row). After 10 minutes of treatment, *n*- and *t*-butanol were washed out (WO). Cells were imaged for 24 minutes in total at 1 frame every 6.6 seconds. Scale bar represents 5 µm.

PLD activity has been shown to control actin remodeling and podosome formation in mouse megakaryocytes and macrophages^27,28^. Therefore, we first investigated whether PLD-dependent PA production is involved in podosome maintenance in primary human monocyte-derived DCs. To block PA production, we took advantage of the ability of the primary alcohol *n*-butanol to compete with water in the PLD-mediated conversion of PC to PA, generating inactive phosphatidyl alcohols instead of PA^42,43^. DCs were allowed to adhere overnight onto glass coverslips and incubated with increasing concentrations of *n*-butanol and fixed at different time points. To evaluate the presence of podosomes, DCs were labeled for actin and vinculin, imaged by fluorescence microscopy and the percentage of podosome containing cells was calculated (Fig. 1a,b). This quantification showed that the addition of *n*-butanol significantly reduced the number of cells with podosomes in a dose- and time-dependent manner (Fig. 1b). Importantly, addition of *tert*-butanol (*t*-butanol), a butanol isomer that is not a PLD substrate, shows little non-significant effect on podosome loss, even after prolonged incubation with high doses (Figs 1a,b and S1), strongly suggesting that the inhibitory effect of *n*-butanol on podosome formation is specifically mediated by PLD activity. To observe the dynamic effect of defective PA production on podosome formation, we performed time-lapse imaging of DCs expressing LifeAct-GFP treated with either *n*-butanol or *t*-butanol (Fig. 1c and Supplementary Movies S1 and S2). We observed that addition of *n*-butanol rapidly resulted in complete podosome dissolution. After *n*-butanol washout, podosomes quickly reformed, indicating that *n*-butanol reversibly affects the ability of DCs to assemble podosomes (Supplementary Movie S1). As expected, *t*-butanol had no effect on the percentage of podosome-forming cells, indicating that the effect on podosome dissolution is not due to lipophilic membrane properties altered by these primary alcohols (Fig. 1c and Supplementary Movie S2).

Podosome formation is mediated by a cascade of events triggered by signals from integrins, receptor tyrosine kinases or G protein-coupled receptors and followed by cell spreading^44,45^. Integrin expression, integrin activation and cell spreading are therefore important factors that have been shown to be controlled by PLD in different cell types^46–48^ and might affect ensuing actin-mediated events. We did not find significant differences in β_2_ integrin or Mac-1 (α_M_β_2_) expression as well as total or active β_1_ integrin levels in cells treated or not with *n*-butanol (Fig. S2). Moreover, cell spreading was largely unaffected (Fig. S3), suggesting that integrin engagement and overall cell spreading were not altered by *n*-butanol-mediated PLD inhibition under the conditions tested (Figs S2 and S3). This is also supported by the observation that in the time-lapse movies, the cells did not shrink or detach after *n*-butanol addition (Fig. 1c and Supplementary Movie S1). Thus, our findings indicate that PLD-dependent PA production is required for DCs to assemble and maintain podosomes, under resting conditions.

### PLD2, but not PLD1, controls podosome maintenance in resting DCs

**Figure 2:**
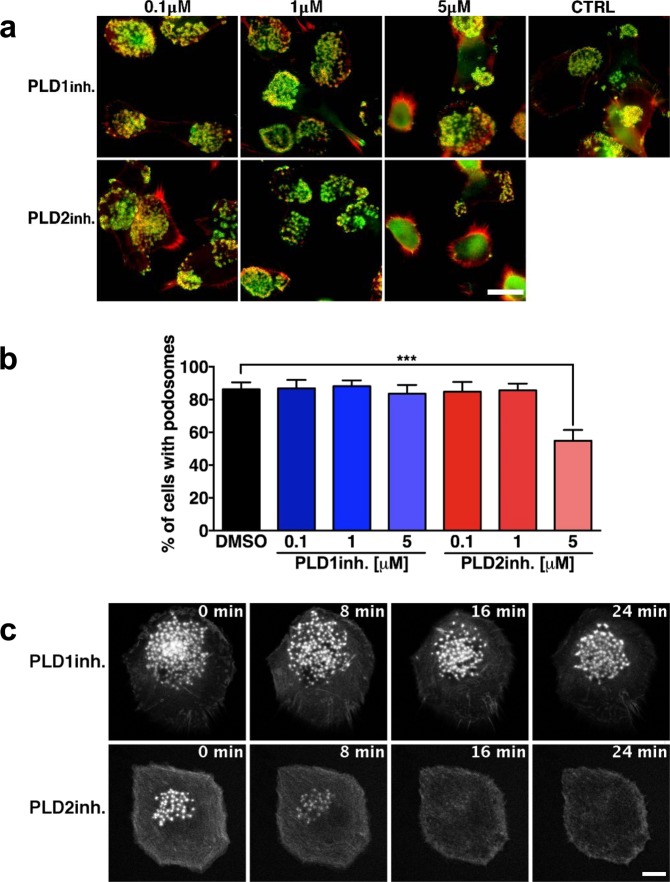
PLD2, but not PLD1, controls podosome maintenance in resting DCs. (**a**) Representative widefield images of DCs treated for indicated times and at indicated doses with PLD1-inh or PLD2-inh. Vinculin is shown in green and F-actin in red. Scalebar represents 20 µm. (**b**) Percentage of cells with podosomes in DCs treated for indicated times and at indicated doses with PLD1-inh or PLD2-inh. Three samples of each experimental condition were prepared for each experiment. A minimum of 25 cells were imaged and analyzed for each sample. Ten independent experiments were performed. Bars represent mean with SEM. Statistical analysis was tested with one-way ANOVA with post-hoc Bonferroni’s multiple comparisons test. Adjusted P-values: ***0.0003. (**c**) Representative confocal images of live cell imaging of DCs transfected with LifeAct-GFP. After 6 minutes of imaging, DCs were treated with 5 µM PLD1-inh (upper row) or PLD2-inh (lower row). Cells were imaged for 25 minutes in total at 1 frame every 4.6 seconds. Scale bar represents 5 µm.

Even though *n*-butanol is commonly used as a PA scavenger, it is not a specific tool to discriminate between the involvement of PLD1 and PLD2 isoforms. Our next aim was therefore to determine whether the PA-dependent podosome formation in resting DCs was mediated by PLD1 or PLD2, which are both expressed in these cells (Fig. S4). Next, we used the pharmacological inhibitors VU 0359595 (PLD1-inh) and VU 0364739 (PLD2-inh) to selectively inhibit PLD1 and PLD2, respectively^49–51^. DCs were left to adhere overnight onto glass coverslips and treated for 10 minutes with different doses of PLD1-inh or PLD2-inh. DCs were subsequently fixed, stained for actin and vinculin and imaged by fluorescence microscopy to evaluate podosome formation (Figs 2a,b and S1). As quantified in Fig. 2b, we found a significant reduction of the percentage of cells with podosomes after the addition of 5 μM PLD2-inh, whereas PLD1 inhibition did not change the percentage of podosome-forming cells. Analysis on cell spreading and integrin expression showed no reduction of integrin surface levels (Fig. S2) and no reduction of cell area (Fig. S3) upon treatment with 5 μM of PLD2-inh. These data suggest that surface integrin levels and cell spreading capability are not affected by PLD1-inh and PLD2-inh and can therefore not be responsible for the reduced percentage of cells with podosomes. Time-lapse imaging of DCs expressing LifeAct-GFP and treated with PLD1-inh and PLD2-inh confirmed the results obtained with fixed cells (Fig. 2c and Supplementary Movies S3 and S4). Altogether these results suggest that PLD2, but not PLD1, controls podosome maintenance in resting DCs.

### Both PLD1 and PLD2 activity contributes to adhesion-induced *de novo* podosome formation

**Figure 3:**
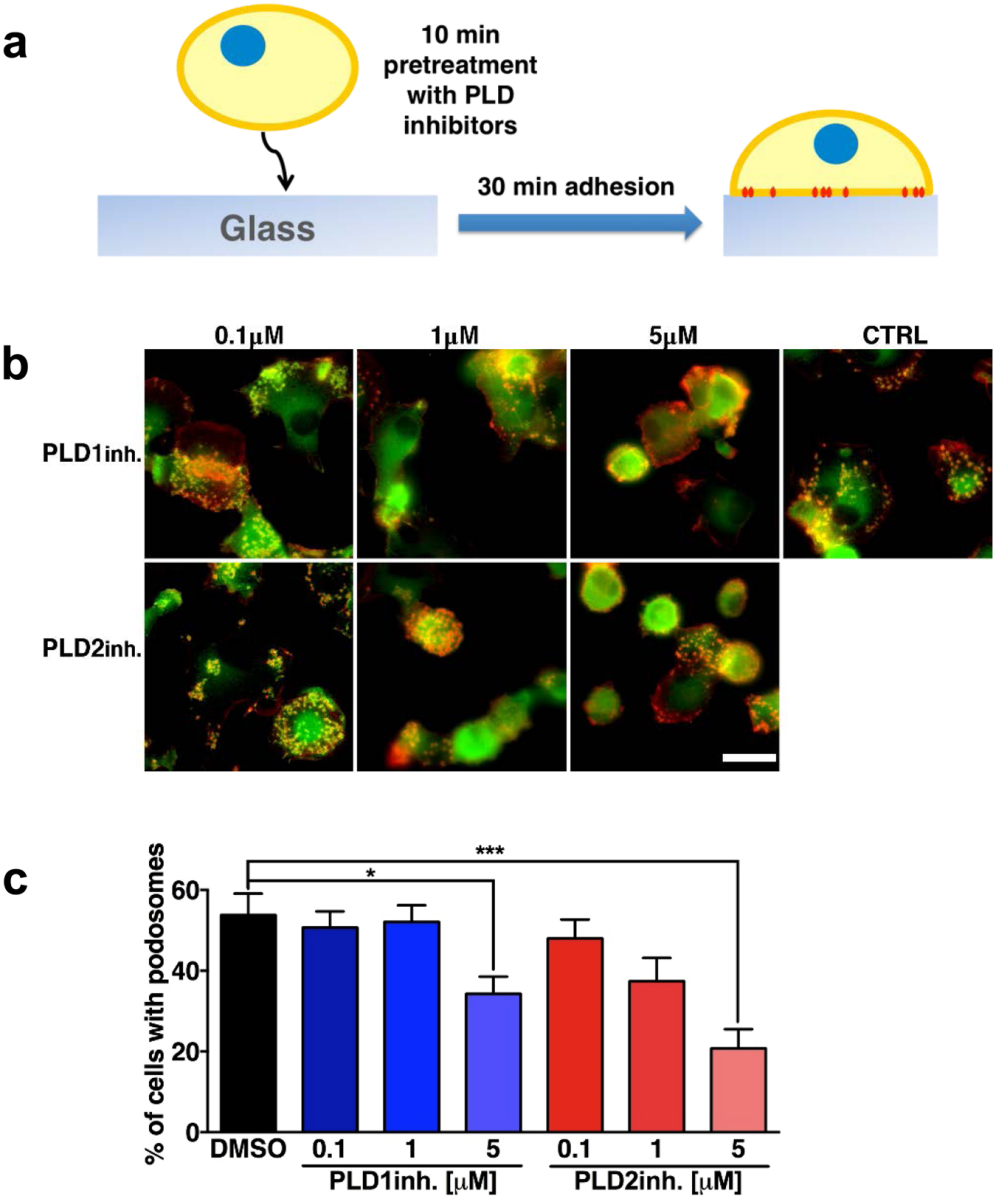
Both PLD1 and PLD2 activity contributes to adhesion-induced *de novo* podosome formation. (**a**) Experimental design scheme. (**b**) Representative widefield images of DCs pretreated for 10 minutes at indicated doses with PLD1-inh or PLD2-inh and subsequently seeded for 30 minutes on glass coverslips. Vinculin is shown in green and F-actin in red. Scale bar represents 20 µm. (**c**) Percentage of cells with podosomes in DCs pretreated for 10 minutes at indicated doses with PLD1-inh or PLD2-inh and subsequently seeded for 30 minutes on fibronectin-coated glass coverslips. Three samples of each experimental condition were prepared for each experiment. A minimum of 25 cells were imaged and analyzed for each sample. Eleven independent experiments were performed. Bars represent mean with SEM. Statistical significance was tested with one-way ANOVA with post-hoc Bonferroni’s multiple comparisons test from. Adjusted P-values: *0.0287, ***0.0003.

Our data demonstrate a role for PLD2 in controlling podosome maintenance in resting DCs, which is in line with the notion that PLD2 is constitutively active as housekeeping enzyme^24,52–54^. Since PLD1 becomes activated by signaling events, such as integrin activation^46,55,56^, we hypothesized that PLD1 may play a role in adhesion-induced *de novo* podosome formation. To test this, we incubated DCs in suspension with or without PLD1-inh or PLD2-inh and subsequently allowed them to adhere to a coverslip (Fig. 3a). To note, we found 30 minutes to be optimal to detect sufficient events of adhesion-induced *de novo* podosome formation (Fig. S5). After 30 minutes, cells were fixed and labeled for vinculin and F-actin and imaged by fluorescence microscopy (Figs 3b,c and S1). We quantified the percentage of DCs forming podosomes in the presence and absence of the PLD inhibitors. Differently from what we observed in resting DCs, *de novo* podosome formation in DCs that were stimulated to adhere appeared to be strongly impaired not only upon PLD2 inhibition, but also upon PLD1 inhibition (Fig. 3b,c). Quantification of cell area after 30 minutes of adhesion under the same conditions showed that PLD2 inhibition and PLD1 inhibition did not affect cell spreading ability (Fig. S3). These data indicate that both PLD1 and PLD2 contribute to adhesion-induced *de novo* podosome formation and strongly suggest that the signaling events that control podosome maintenance and *de novo* formation are different.

### PLD1 and PLD2 both control synchronized podosome reformation

**Figure 4:**
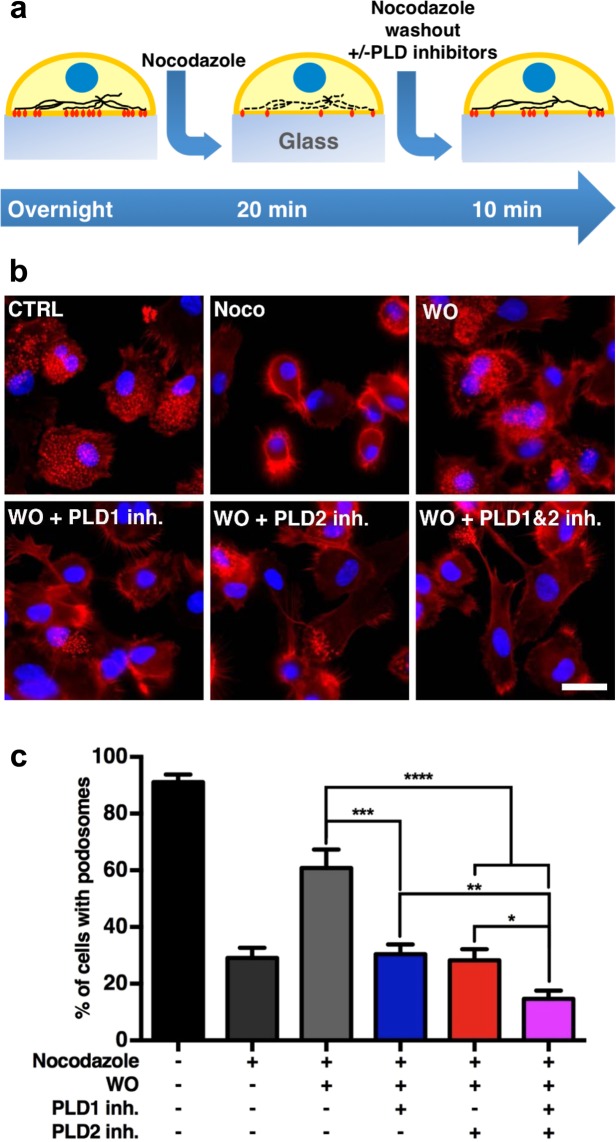
PLD1 and PLD2 both control synchronized podosome reformation. (**a**) Experimental design scheme. DCs were treated with 5 µM nocodazole for 20 minutes. Then nocodazole was replaced (washed out; WO) by medium with or without PLD1-inh or PLD2-inh and cells were treated for additional 10 minutes cells with or without 5 µM PLD1-inh or PLD2-inh. (**b**) Representative widefield images of DCs treated as indicated in panel a. F-actin is shown in red and the nucleus in blue. Scale bar represents 20 µm. (**c**) Percentage of cells with podosomes in DCs treated as indicated in panel a. Three samples of each experimental condition were prepared for each experiment. A minimum of 25 cells were imaged and analyzed for each sample. Eight independent experiments were performed. Bars represent mean with SEM. Statistical significance was tested with one way ANOVA with post-hoc Bonferroni’s multiple comparisons test. Adjusted P-values: ***0.0004 and ****0.0001.

So far, our results indicate a general involvement of PLD2 in podosome maintenance and a specific role for PLD1 activity in mediating podosome *de novo* formation triggered by adhesion. To further confirm the involvement of PLD1 in *de novo* podosome formation, we treated adherent DCs with the microtubule disrupting compound nocodazole, which results in podosome disappearance. As already reported^57,58^, nocodazole treatment followed by thorough washout results in synchronized *de novo* podosome formation (Fig. 4a). Adding PLD inhibitors during nocodazole washout allowed us to further study PLD involvement in *de novo* podosome formation. After the pharmacological treatments, DCs were fixed and labeled for F-actin and the nucleus and imaged by fluorescence microscopy (Fig. 4b). Quantification of the number of cells with podosomes under the different conditions was performed (Fig. 4c). As expected, nocodazole treatment reduced the number of cells with podosomes from 90% to 30%, and the subsequent washout of nocodazole significantly restored the percentage of cells forming podosomes to almost 60% (Fig. 4c). Interestingly, addition of both PLD1-inh and PLD2-inh during nocodazole washout completely prevented synchronized reformation of podosomes, indicating that in this situation PLD1 and PLD2 have an independent and non-redundant function (Fig. 4c). This is further substantiated by the fact that simultaneous inhibition of PLD1 and PLD2 showed an even greater reduction of podosome reformation (Fig. 4c). These inhibitory effects on podosome formation cannot be attributed to differences in cell spreading, as this was similar for all treatments (Fig. S3). These results further confirm the importance of PLD1 specifically during the initial phases of *de novo* podosome formation, during which PLD2 also plays an important role.

### PLD1 and PLD2 both control fMLP-stimulated podosome formation

**Figure 5:**
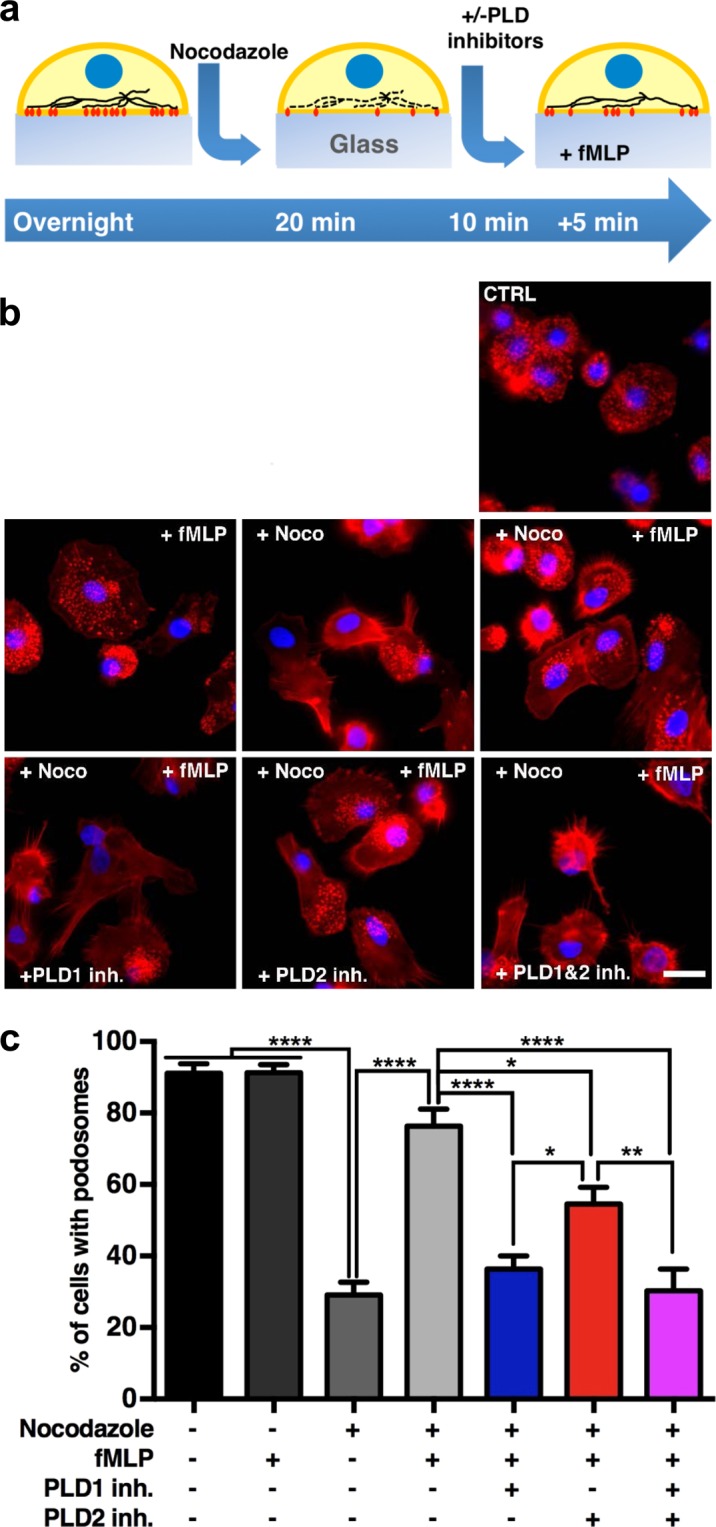
PLD1 and PLD2 both control fMLP-stimulated podosome formation. (**a**) Experimental design scheme. DCs were treated with 5 µM nocodazole for 20 minutes. Then nocodazole was replaced (washed out; WO) by medium with or without PLD1-inh or PLD2-inh and cells were treated for an additional 10 minutes with or without 5 µM PLD1-inh or PLD2-inh. Finally, fMLP was added. Cells were treated with 1 µM fMLP for 5 minutes in the presence or absence of PLD1-inh or PLD2-inh. (**b**) Representative widefield images of DCs treated as indicated in panel a. F-actin is shown in red and the nucleus in blue. Scale bar represents 20 µm. (**c**) Percentage of cells with podosomes in DCs treated as indicated in panel a. Three samples of each experimental condition were prepared for each experiment. A minimum of 25 cells were imaged and analyzed for each sample. Six independent experiments were performed. Bars represent SEM. Statistical significance was tested with one-way ANOVA with post-hoc Bonferroni’s multiple comparisons test. Adjusted P-values: *0.0404, *0.0154 and ****0.0001.

The potent leukocyte chemoattractant *N*-formyl-methionyl-leucyl-phenylalanine (fMLP) has been shown to induce podosome formation in macrophages^59^ and to control cell migration in DCs^60^. Since PLD1 has been shown to be regulated by fMLP in mouse and human neutrophils^55,61,62^, we investigated whether fMLP-induced PLD1 activation leads to podosome formation in human DCs (Fig. 5a). For this, podosomes of adherent DCs were first dissolved by a pretreatment with nocodazole and subsequently cells were stimulated with fMLP in the absence and presence of the PLD1-inh or PLD2-inh (Fig. 5b,c). Addition of fMLP to resting DCs did not increase the number of cells with podosomes as already over 90% of immature DCs make podosomes in the untreated control situation (Fig. 5b,c). In contrast, when fMLP was added to DCs treated with nocodazole, we observed almost complete restoration of the percentage of podosome forming cells, indicating that fMLP is able to induce podosome formation even in the presence of nocodazole. When fMLP was added after nocodazole treatment in the presence of PLD1-inh, podosome *de novo* formation was totally impaired, whereas treatment with PLD2-inh reduced, but did not completely prevent, fMLP-induced podosome formation (Fig. 5c). Finally, simultaneous inhibition of PLD1 and PLD2 showed an inhibitory effect on podosome reformation similar to treatment with PLD1-inh only. Quantification of the average number of podosomes per cell showed that untreated cells as well as fMLP treated cells made an average of 100 podosomes/cell, indicating that unlike previous observations in macrophages, fMLP does not increase the number of podosomes in human DC (Fig. S6). Moreover, the few cells that made podosomes after PLD1-inh treatment display only a slightly lower number of podosomes per cell (Fig. S6). Quantification of cell size analysis under the same conditions showed once again that cell spreading was unaffected (Fig. S3). These data demonstrate for the first time that fMLP induces *de novo* podosome formation in human DCs and that PLD1 plays a significant role in this process. Since PLD2 also contributes, these data suggests that PLD1 is necessary, but not sufficient, to mediate the regulation of fMLP-induced podosome formation.

### PA microdomains colocalize with podosomes and show concerted dynamics with actin cores

**Figure 6:**
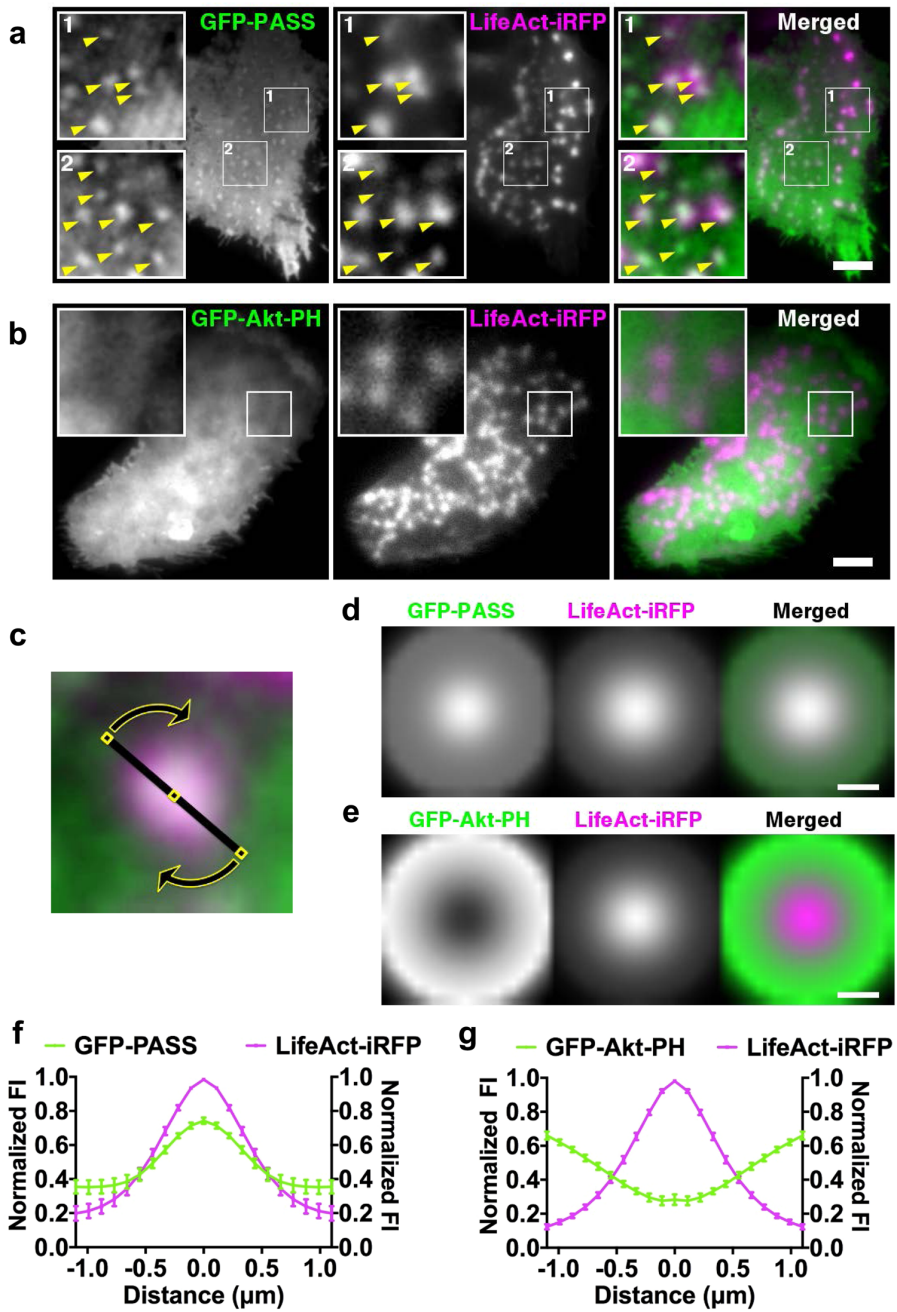
PA is detected at podosomes. (**a**,**b**) Representative TIRF images of live cell imaging of DCs transfected with GFP-PASS (**a**) or GFP-Akt-PH (**b**), in combination with LifeAct-iRFP. In the merged image LifeAct-iRFP is shown in magenta and GFP-PASS (**a**) or GFP-Akt-PH (**b**) is shown in green. Boxes depict enlargements of a podosome-rich area where arrowheads indicate single podosomes. Scalebar represents 5 µm. (**c**) Representative TIRF microscopy image of a 25 by 25 pixel area containing a single podosome. Fluorescence intensity profile analysis for a single podosome was performed by averaging the intensity profiles obtained along the indicated line ROI (20 pixels), centered in the core of the podosome, projected over 360 degrees. (**d**,**e**) 360° average fluorescence profile of a single podosome in DCs transfected with GFP-PASS and LifeAct-iRFP (**d**) or GFP-Akt-PH and LifeAct-iRFP (**e**). >100 podosomes in total (from 10 cells) were analyzed for both transfections. (**f**,**g**) Average fluorescence intensity profile of a single podosome in DCs transfected with GFP-PASS and LifeAct-iRFP (**f**) or GFP-Akt-PH and LifeAct-iRFP (**g**). >100 podosomes in total (from ten cells) were analyzed for both transfections. Data points/lines indicate mean with SD.

To understand how PLD regulates podosome formation, we examined the production of PA at sites of podosome formation using a GFP-tagged PA biosensor with superior sensitivity (GFP-PASS)^41^. GFP-PASS was transfected into human primary DCs together with LifeAct-iRFP, to simultaneously capture the spatiotemporal dynamics of PA and actin at the plasma membrane by Total Internal Reflection Fluorescence (TIRF) microscopy (Supplementary Movie S5). Visual inspection of these time-lapse movies revealed the presence of small and dynamic GFP-PASS microdomains within individual podosome actin cores that occupy, according to our measurements, a circular area of about 700 nm on average (Fig. 6a and Supplementary Movie S5), suggesting the presence of PA at these locations. By contrast, the phosphatidylinositol (3,4,5)-trisphosphate (PIP_3_) sensor GFP-Akt-PH was not enriched at LifeAct-iRFP-labeled podosomes in DCs (Fig. 6b and Supplementary Movie S6). To further strengthen this observation, we averaged and compared the radial fluorescent intensity profiles from a 360° region of interest (ROI) of both GFP-PASS and LifeAct-iRFP from hundreds of individual podosomes from multiple cells and confirmed that PA and actin colocalize at the site of podosomes (Figs 6c,d,f and S7). By contrast, although GFP-Akt-PH clearly localized to the plasma membrane, the fluorescence signal of this PIP_3_ sensor did not show the same enrichment observed for PASS-GFP at the podosome core (Figs 6e,g and S7).

**Figure 7:**
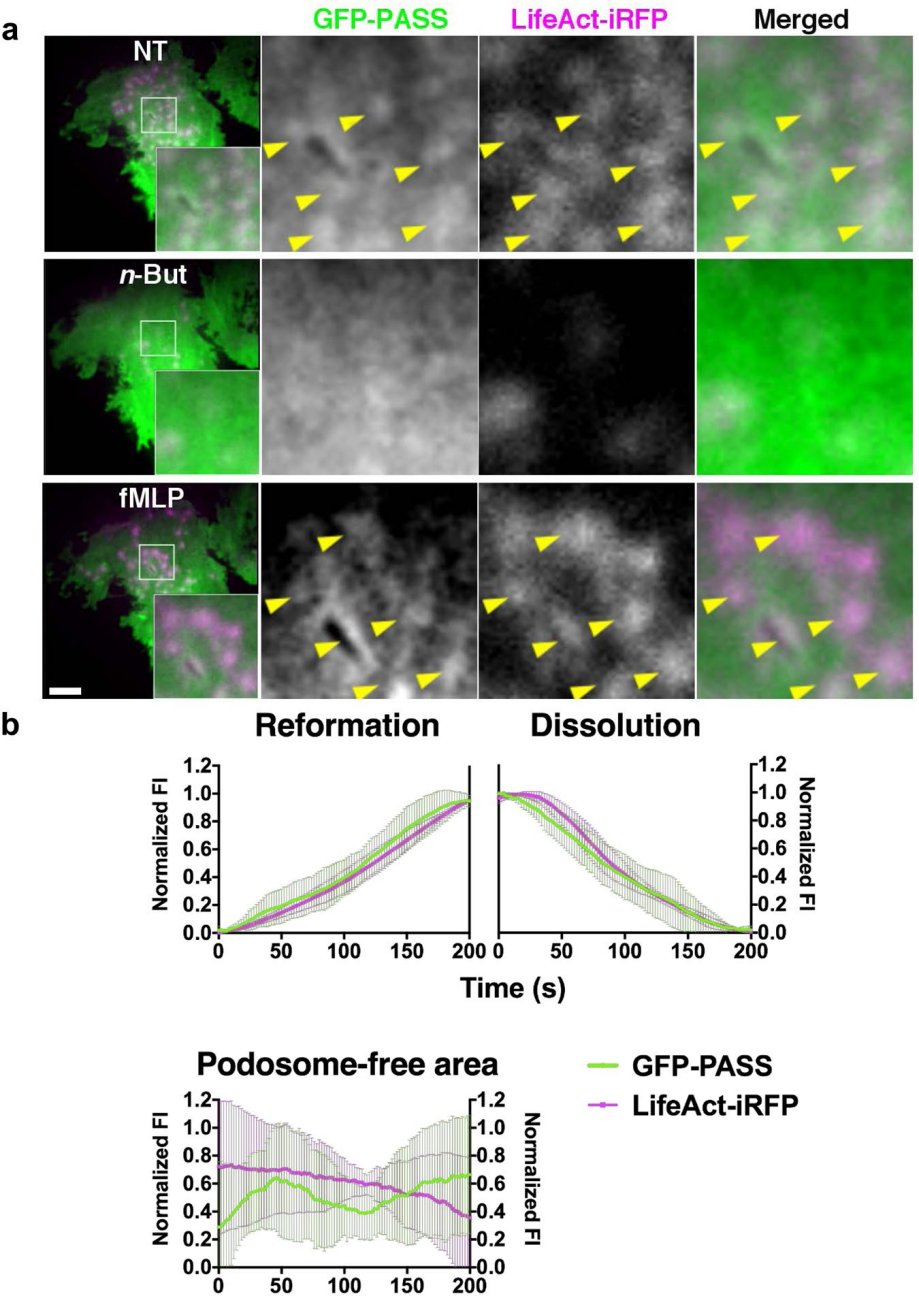
PA enrichment correlates with core actin polymerization. (**a**) Representative images of TIRF live cell imaging of DCs transfected with GFP-PASS and LifeAct-iRFP, before treatment (NT; top row), subsequently treated for 10 minutes with 25 mM *n*-butanol (*n*-But; middle row) and finally stimulated for 10 minutes with 1 µM fMLP (fMLP; bottom row). In the merged image LifeAct-iRFP is shown in magenta and GFP-PASS is shown in green. In the magnified square arrows indicate podosomes or PA microdomains. Cells were imaged for 39 minutes in total at 1 frame every 2 seconds. Scalebar represents 5 µm. (**b**) Average fluorescence intensity profile of a single podosome in DCs pretreated with *n*-butanol and stimulated with fMLP as indicated in panel a. As a control, a podosome-free area in the cell was analyzed. Cells were imaged for 15 minutes in total at 1 frame every 2 seconds. Fortyfive podosomes in total from 4 cells were analyzed for both conditions. Data points indicate mean with SEM.

Next we investigated whether inhibition of PLD1 or PLD2 interfered with PA microdomain formation at podosomes. Addition of PLD2-inh to resting DCs induced loss of the GFP-PASS signal and concomitant podosome dissolution (Fig. S8 and Supplementary Movie S7). In contrast, PLD1 inhibition did not affect podosome maintenance and PA microdomains (Fig. S8 and Supplementary Movie S8), in line with our previous observations that PLD2, but not PLD1, is involved in podosome turnover in resting DCs. In order to understand whether PA enrichment correlates with core actin polymerization, we analyzed podosome formation and dissolution dynamics in DCs co-transfected with GFP-PASS and LifeAct-iRFP and stimulated with fMLP. We first treated the cells with *n*-butanol to dissolve podosomes and then stimulated them with fMLP, while recording images every 2 seconds by TIRF microscopy (Fig. 7a and Supplementary Movie S9). We subsequently plotted the average fluorescence intensity of both GFP-PASS and LifeAct-iRFP over time during formation and dissolution of multiple individual podosomes and found a complete correlation between PA and actin levels (Fig. 7b). No specific correlation was observed in cell areas with no podosomes (Fig. 7b). Altogether our results demonstrate for the first time that PA accumulates at podosome sites in a PLD activity-dependent manner and that these PA microdomains dynamically correlate with actin during podosome formation and dissolution.

### PLD1 and PLD2 activity is required for matrix degradation

**Figure 8:**
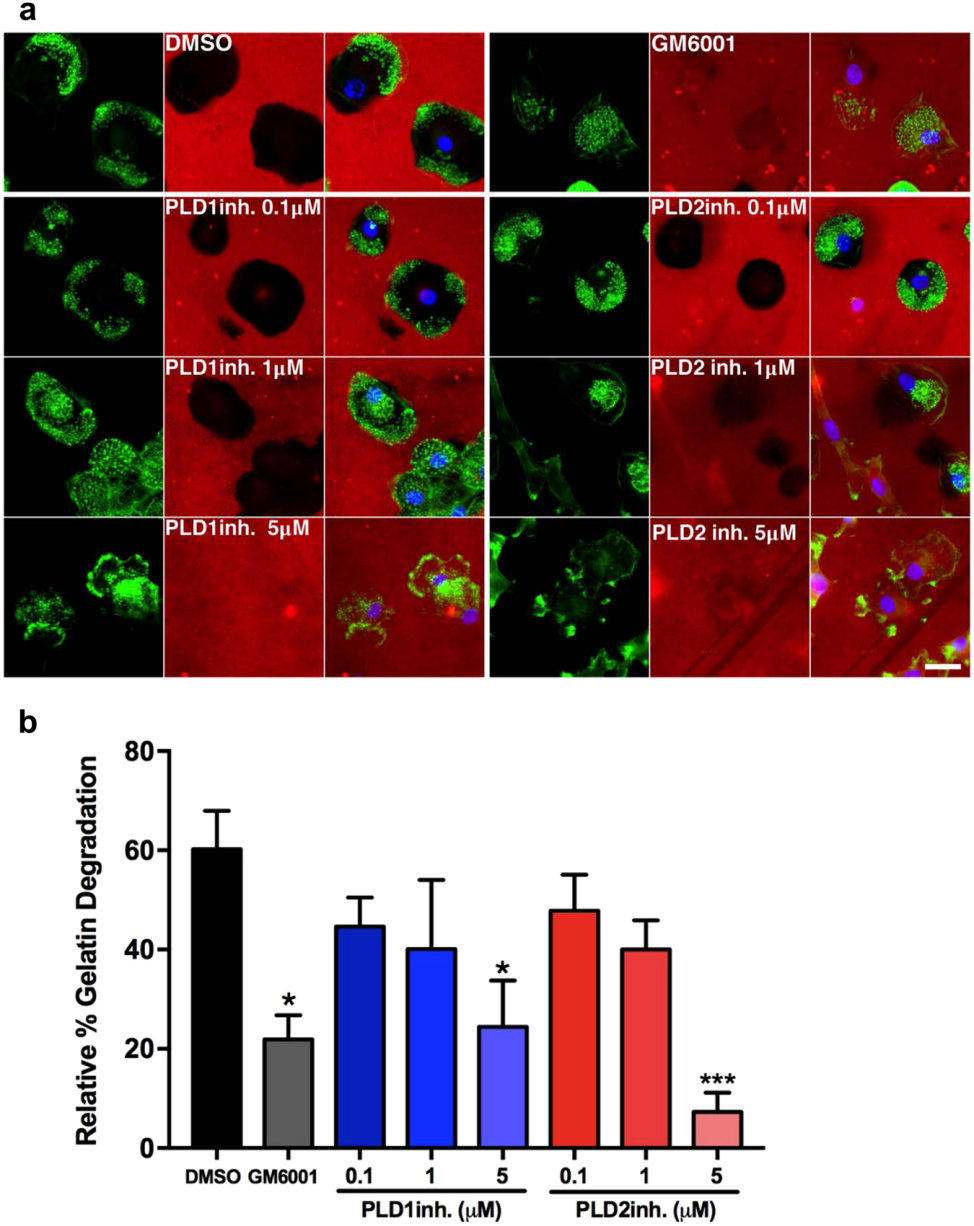
PLD1 and PLD2 activity is required for matrix degradation. (**a**) Representative widefield images of DCs seeded on Rhodamine-B-conjugated gelatin, treated with 5 µM MMP-inhibitor GM6001, or with indicated doses of PLD1-inh or PLD2-inh overnight. F-actin is shown in green, nuclei are shown in blue and gelatin is shown in red. Scalebar represents 20 µm. (**b**) Relative percentage of degraded gelatin in DCs treated at indicated doses with PLD1-inh or PLD2-inh or with 5 µM GM6001. Three samples of each experimental condition were prepared for each experiment. A minimum of 50 cells were imaged and analyzed for each condition. Four independent experiments were performed. Bars represent mean with SEM. Statistical significance was tested with one-way ANOVA with post-hoc Bonferroni’s multiple comparisons test. Asterisks indicate statistical significant differences compared to non-treated control (NT). Adjusted P-values: 0.0213 < * > 0.0129, ***0.0005.

PA has been shown to be involved in several cellular phenomena including actin polymerization, vesicle transport and extracellular matrix degradation^63,64^. Since matrix degradation is an important function of podosomes^65^ and PLD activity has been associated with matrix degradation in migrating cancer cells^64,66^, we sought to investigate the effect of pharmacological inhibition of PLD1 and PLD2 on matrix degradation in DCs. After DCs were seeded on gelatin-Rhodamine-B-coated glass coverslips for three hours, cells were treated overnight with different doses of PLD1-inh or PLD2-inh before they were fixed and stained (Fig. 8a). Analysis of percentage of matrix degradation underneath the cells shows that, albeit to a different extent, both PLD1 and PLD2 are involved in matrix degradation (Fig. 8b). Treatment with PLD1-inh reduced the digested area to a level comparable to the negative control, treatment with general metalloprotease inhibitor GM6001 (20%), and treatment with PLD2-inh reduced the digested area under the cells even further (7%). Matrix degradation was not accompanied by podosome dissolution in DCs treated with PLD1-inh as it was in DCs treated with PLD2-inh, indicating alternate functions for the two isoforms. Altogether, our data demonstrate that PLD1 and PLD2 are not only essential signaling molecules for podosome formation and maturation, but that they also play a key role in controlling matrix degradation in human DCs.

## Discussion

The aim of this study was to investigate the role of PLD1, PLD2, and their catalytic product PA, in regulating podosome formation and maintenance in human DCs. We first demonstrated that PA production is essential for the maintenance of podosomes in resting DCs. Next, we revealed a differential role for PLD1 and PDL2 isoforms in podosome formation, with only PLD2 responsible for podosome formation and maintenance in resting cells and both PLD1 and PLD 2 contributing to *de novo* podosome formation induced by either adhesion, microtubule reformation or fMLP signaling. Importantly, we also found that small PA microdomains specifically accumulate at podosomes in a coordinated fashion with the actin core. Finally, we demonstrated that both PLD isoforms are important for matrix degradation.

In murine RAW264.7/LR5 macrophages, PLD1 has been shown to undergo a rapid and transient activation, whereas PLD2 exhibited a slower but sustained activity^24^. Our data support similar activation kinetics for PLD1 and PLD2 in human DCs. PLD2 activity is responsible for podosome maintenance for prolonged adhesion times (Fig. 2, Movie S3), whereas PLD1 activity specifically contributes to *de novo* podosome formation occurring at the onset of induced cell adhesion (Fig. 3 and Movie S5), or after chemoattractant signaling or after microtubule reformation (Figs 4 and 5). In primary mouse macrophages, PLD1 knockout resulted in fewer but larger podosomes, whereas PLD2 knockout led to reduced podosome size^28^. Moreover, non-redundant, partially redundant, and fully redundant roles for PLD1 and PLD2 have been reported^28,67,68^. Interestingly, in cells treated with nocodazole, PLD2 and PLD1 inhibition reduced the number of podosome forming/containing cells to a comparable extent, while their simultaneous inhibition clearly showed an additive effect. These results suggest a non-redundant, concurrent mechanism in which both enzymes contribute to podosome formation.

PLD activation following integrin engagement or agonist stimulation with a wide variety of ligands acting via G-protein-coupled receptors or receptor tyrosine kinases has been observed in various cell types^69–71^. The potent chemoattractant fMLP has been used as activator of signaling pathways mediated by Rho family members, including PLD1^72^, and can mediate actin-based activities, such as cell adhesion, spreading, and the formation of podosomes^59,73^. So far, signaling by fMLP receptors has been shown to be mediated mainly by PLD1^55,61,62,72^. Our data confirm the role of PLD1 in mediating fMLP-induced signaling, as PLD1-inh strongly prevented podosome reformation in presence of fMLP. Interestingly, we also found that fMLP-induced podosome formation is mediated by PLD2 (Fig. 5). Finally, our results indicate that fMLP is able to induce podosome formation in the absence of microtubules, which normally are essential for podosome formation^74^ (Fig. 5). This is in line with reports showing that fMLP can directly activate Cdc42 or Rac1 in the context of actin nucleation and branching in human neutrophils^75^.

Both PLD1 and PLD2 have been shown to enhance secretion of soluble matrix metalloproteinases (MMPs) to degrade surrounding extracellular matrix and facilitate cellular movement^76–78^. In addition, it has recently been found that PLD2-mediated PA production promotes the plasma membrane targeting of MT1-MMP through binding to the motor protein kinesin-1^64^. Our data not only substantiate previous evidence on the role of PLD1 and PLD2 in the release of MMPs, but also show for the first time that podosome presence and matrix degradation can be unlinked from each other by targeting a specific PLD isoform (Fig. 8). In DCs under resting conditions, PLD1 may only control the release of MMPs, while PLD2 may control both the release of MMPs and podosome formation, as treatment with PLD2-inh resulted in lower matrix degradation in absence of podosomes, but DCs treated with PLD1-inh still were able to form podosomes, but also showed less matrix degradation. Recently, MT1-MMP islets have been identified as memory devices to enable efficient and localized podosome reformation, which ultimately ensures coordination of matrix degradation and cell invasion^65^. Based on our results, we propose that inhibition of PLD2, but not PLD1, could prevent MT1-MMP islet formation and subsequent podosome reformation. Further investigation is necessary to directly prove the effect of specific PLD isoform inhibition on the location and activity of both soluble MMPs and membrane-bound MT1-MMP.

Previous work showed that PA is accumulated in microdomains at the plasma membrane/vesicle fusion site during exocytosis^40^. With TIRF microscopy, we demonstrated accumulation of PA sensor at the site of podosome formation (Fig. 6 and Movie S5). Since classical Spo20-derived PA sensors are under debate for selectivity^79^ we rather preferred to use the more selective PASS-GFP sensor. Although it is still derived from Spo20, it has been edited to increase the enrichment to the membrane and demonstrated increased selectivity to PA compared to PIP_2_ or PIP_3_^41^. With this construct, we have been able to demonstrate the existence of small PA microdomains specifically accumulating at the protrusive podosome core. These findings strongly suggest that PA functions as a signaling lipid to induce actin polymerization required for podosome formation. Surprisingly, we found that GFP-Akt-PH, a fluorescent probe that binds PI(3,4)P_2_ and PI(3,4,5)P_3_, localizes diffusely at the cell membrane and in much smaller measure in the podosome around. These results are in contrast with a recent study where GFP-Akt-PH was used to image time-dependent PI(3,4,5)P_3_ production during podosome formation in fibroblasts seating on lipid bilayers functionalized with freely moving RGD peptides and imaged using spinning disk confocal microscopy^80^. This discrepancy reflects the different dependence on PI(3,4,5)P_3_ for podosomes in different cell types, and suggests that this lipid is not essential for podosome formation in DCs. By contrast, PA has been reported to be critical for podosomes in multiple cell types, such as megakaryocytes, macrophages and now in DCs, suggesting it is an essential signaling lipid for podosome formation.

Here we showed that actin polymerization and PA microdomain presence are strongly connected (Fig. 7 and Movie S9). The exact kinetics of these two dynamic processes remain to be established, since attempts to temporally dissect these two processes by TIRF microscopy using very short acquisition times ( < 2 seconds) led to photobleaching and phototoxicity. The use of the PA biosensor fused with a more photostable and bright fluorescent protein might allow us to address this question in the future. Since pharmacological inhibition of PLD activity reduces the number of podosomes, we propose that PA is critical to podosome formation. It is also likely that PA and actin polymerization form a feedforward loop to promote podosome formation. Here, we propose PA as pivotal phospholipid able to control cell protrusion and matrix degradation through its ability to accumulate in dense microdomains that act as signaling platforms for local actin polymerization. Moreover, PA accumulation might contribute to locally shape membrane curvature at sites of podosome formation, thus contributing to attract some of the membrane curvature binding proteins that are known to localize at podosomes^81–83^.

How do PLD1 and PLD2 together control podosome assembly? Considering the dynamic protrusive behavior of individual podosomes^84^ and mesoscale coordination of podosome clusters^58^, a tight spatiotemporal regulation of several Rho family members is expected to control local changes in podosome architecture and dynamics^85^. In this complex scenario, our data support different roles for PLD1 and PLD2 in controlling podosome formation under resting conditions or upon acute signaling (Figs 2 and 3). One possible explanation would be existence of a Rho, Arf, PLD1, PLD2 and PIP5K signaling module, which has been already postulated^69,86,87^. Indeed Rho family members have been demonstrated to control podosome formation^1,88,89^, podosome distribution^90^ and, in the case of RhoA, both assembly and dissolution^1,91,92^. In addition, PLD1- and PLD2-generated PA is reported to activate and directly regulate PIP5K activity^38,93,94^, and PLD2 can recruit PIP5K to the membrane^95^. Finally, PIP5K has been show to localize at podosome rings in osteoclasts^96^. Since our data show that both PLD1 and PLD2, albeit with some differences, promote podosome formation in a non-redundant concurrent manner (Fig. 4c), it is plausible to speculate that a signaling module composed by all these proteins might exist to tightly regulate podosome spatiotemporal organization. Accordingly, PLD1 and PIP5K, which are both under the control of external stimuli, such as adhesion, chemokines and chemotactic factors mediated by Arf and Rho^87^, could drive *de novo* podosome formation following immediate adhesion. The requirement of PLD1 in the early events of podosome formation is possibly necessary because PLD2 activity only would not be sufficient to reach the local threshold of PA concentration necessary to induce *de novo* podosome formation. At steady state, when podosomes are mature, PLD1 gets inactive and only PLD2, probably with the help of other signaling partners such as PIP5K, induces PA-PI(4,5)P_2_ loop oscillations driving podosome dynamic behavior.

As discussed above, our data support similar activation kinetics, but a difference in short-lived vs. sustained activity, of PLD1 vs. PLD2. Comparing formation and dissolution of podosomes and PA microdomains in PLD1 and PLD2-deficient cells transfected with PASS-GFP would be able to shed more light on kinetics of PLD1 and PLD2 activity in these processes. Moreover, another question that needs to be addressed is whether PLD1 and PLD2 are differentially localized in podosome-forming cells and would be able to generate different pools of PA. To perform such experiments, transfection of catalytically-inactive mutants of PLD1 and PLD2 isoforms and/or siRNA-based studies need to be performed. However, both are under debate for the interpretation of the effects they may cause. On the one hand, dominant-negative alleles could displace endogenous PLD proteins or could sequester other signaling factors. On the other hand, RNAi decreases, but does not eliminate, expression, has a limited window of activity, and can cause cellular stress or off-target effects^20^. A possible solution could be provided by making use of the novel CRISPR/Cas9 technology.

Overall, our study demonstrates the dynamic interplay between membrane lipid remodeling and local induction of actin polymerization that mediates the formation of protrusive podosomes. In this context, PLD1, PLD2 and their metabolite PA are emerging as part of a signaling system for the control of adhesion and migration of human tissue-resident DCs.

## Materials and Methods

### Preparation of human DCs

DCs were generated from peripheral blood mono-nuclear cells (PBMCs) as described previously^97^. Shortly, buffy coats or leukapheresis products from healthy individuals were obtained from Sanquin Bloodbank, Nijmegen the Netherlands, and PBMCs were isolated by density gradient centrifugation using Lymphoprep (Cat# AXI-1114547, Axis-Shield). To generate immature monocyte-derived DCs (moDCs), the plastic-adherent monocyte fraction was cultured for 5 to 6 days in RPMI 1640 medium (Cat# 42401018, Thermo Fisher Scientific) supplemented with fetal calf serum (FCS, Cat# 10270106, Gibco, Thermo Fisher Scientific), 1 mM Ultra-glutamine (Cat# BE17-605E/U1, Lonza), antibiotics (Antibiotic-Antimycotic (AA) Cat# 15240062, from Gibco, Thermo Fisher Scientific, containing 100 U/ml penicillin, 100 mg/ml streptomycin and 0.25 mg/ml amphotericin B), IL-4 (300 U/ml, Cat# 1403-050, Cellgenix) and GM-CSF (450 U/ml, Cat# 1412-050, Cellgenix) at 37 °C in a humidified, 5% CO_2_-containing atmosphere.

### Antibodies and reagents

The following primary antibodies were used: mouse anti-human vinculin (clone hVIN-1, Cat# V9131, Sigma-Aldrich), mouse-anti-human β_2_ integrins (clone L19; from hybridoma), mouse-anti-human β_1_ integrins (active fraction, clone 12G10; Cat# MAB2247, EMD Millipore), mouse-anti-human β_1_ integrins (total fraction, clone 4B4; from hybridoma) and mouse anti-human Mac-1 (α_M_β_2_ integrin) (clone Bear-1; Cat# MAB4125, Abnova). Secondary antibodies goat-anti-mouse IgG(H + L)-Alexa488 or goat-anti-mouse IgG(H + L)-Alexa647 secondary antibodies were used (respectively Cat#A11029 and Cat#A21235, Thermo Fischer Scientific). Alexa Fluor633- and Alexa Fluor488–conjugated phalloidin (respectively Cat# A22284 and Cat#12379, Thermo Fischer Scientific) was used to stain F-actin and DAPI (Cat# 32670, Sigma-Aldrich) was used to stain the nucleus. *n*-butanol was obtained from Merck (Cat# 1019901000) and *t*-butanol was obtained from Sigma-Aldrich (Cat# 360538), PLD1 inhibitor (Cat# VU0155069) (IC_50 PLD1_ 3.7 nM/IC_50 PLD2_ 6.4 μM), referred to from here and in the text as PLD1-inh, was from Cayman Chemical Company; PLD2 inhibitor (Cat# VU0364739) (IC_50 PLD2_ 20 nM /IC_50 PLD1_ 1.5 μM), referred to from here and in the text as PLD2-inh, was from TOCRIS Biosciences and microtubule disrupting agent Nocodazole was obtained from Sigma-Aldrich (Cat# M1404). Leukocyte chemoattractant *N*-formyl-methionyl-leucyl-phenylalanine (fMLP) was obtained from Sigma-Aldrich (Cat# F3506), Matrix Metalloproteinase (MMP) inhibitor GM6001 was obtained from Calbiochem (Cat# 364205).

### Immunofluorescence

In general, immature moDCs (DCs) day 5 or 6 were seeded onto #1.5 12 mm glass coverslips (Electron Microscopy Sciences) coated with human fibronectin (from human plasma, Cat# 11080938001, Sigma-Aldrich). Three coverslips per condition were prepared. For experiments involving evaluation of podosome steady state formation and maintenance, DCs were left to adhere overnight. The next day, DCs were treated with either *n*-butanol (5, 25 or 50 mM for 1, 5 or 20 minutes) or *t*-butanol (5, 25 or 50 mM for 20 minutes) or left untreated. For specific PLD1 and 2 inhibition, DCs were treated with 0.1, 1 or 5 µM PLD1-inh or PLD2-inh or DMSO (negative control) for 10 minutes. For evaluation of the role of PLD1 and 2 in *de novo* podosome formation, cells were pre-treated with 1 or 5 µM PLD1-inh or PLD2-inh for 10 minutes or left untreated, before they were seeded for 30 minutes on coverslips. For synchronized podosome re-formation, DCs were left to adhere overnight to form mature podosomes. Subsequently cells were treated with 5 µM nocodazole for 20 minutes. Finally, nocodazole was washed out by adding complete medium or complete medium with 5 µM of PLD1-inh or PLD2-inh. Podosome formation was induced in presence of nocodazole, by adding medium containing fMLP with or without PLD1-inh or PLD2-inh, reaching a final concentration of 1 µM fMLP and 5 µM PLD1-inh or PLD2-inh.

In all experiments, after treatment, cells were fixed in 3.7% (w/v) formaldehyde in PBS for 10 minutes. Cells were permeabilized in 0.1% (v/v) Triton X-100 (Cat# 39795.02, SERVA Electrophoresis GmbH) in PBS for 5 minutes and blocked 30 minutes with 2% (w/v) BSA in PBS. The cells were incubated with primary mouse anti-human vinculin antibody in 2% BSA in PBS for 1 hour. Subsequently, the cells were washed with PBS and incubated with Alexa Fluor 488-labeled secondary anti goat anti-mouse (H + L) antibody for 45 minutes. Finally, cells were incubated with Alexa633-conjugated phalloidin for 30 minutes. Finally samples were washed with phosphate buffer prior to embedding in Mowiol (Sigma-Aldrich). Fixed samples for butanol experiments were imaged with a Leica DMRA epi-fluorescence microscope equipped with a 63×/1.3 NA PL APO oil immersion objective. All other fixed cell experiments were imaged with a DMI6000 epi-fluorescence microscope equipped with a 63×/1.4 NA PL APO oil immersion objective.

### Gelatin degradation assay

Gelatin (Cat# G1890, Sigma-Aldrich) was labeled with Rhodamine-B (Cat# R6626, Sigma-Aldrich) as previously described with the exception that free dye was removed by overnight dialysis against PBS^98^. Coverslips were first incubated with 50 μg/ml poly-L-lysine at RT for 20 minutes. After washing 3 times with PBS, dishes were incubated with 0.25% glutaraldehyde for 15 minutes at RT. Again, coverslips were washed 3 times with PBS and then incubated with 25 μg/ml Rhodamine-B conjugated gelatin for 10 minutes at RT. Coverslips were then washed 3 times, sterilized with 70% ethanol and finally washed three times with PBS. Before use, coverslips were blocked with RPMI-1640 medium at 37 °C for at least 30 minutes. DCs were seeded onto gelatin-coated coverslips with a density of ~300,000 cells per well in complete medium (RPMI-1640 containing 10% FCS, 1% Ultraglutamine and 0.5% AA). Cells were incubated overnight at 37 °C and fixed with 1% PFA RPMI-1640 solution at 37 °C for 30 minutes. After washing with PBS, cells were permeabilized with 0.1% Triton X-100 for 5 minutes at RT. Cells were again washed with PBS and then blocked with 2% BSA for at least 30 minutes. To visualize F-actin and vinculin, cells were stained with Alexa488-conjugated phalloidin and mouse anti-human vinculin antibody that was detected by a goat anti-mouse Alexa647-conjugated secondary antibody.

### Transfection and live-cell imaging

For live-cell imaging cells were transfected with LifeAct-RFP only, or LifeAct-iRFP (infrared fluorescent protein) in combination with PASS-GFP or Akt-PH-GFP. Transient transfections were carried out with the Neon Transfection System (Invitrogen). DCs day 5 or 6 were washed with PBS and resuspended in Resuspension Buffer at 5*10^6^ cells per ml. Subsequently, cells were mixed with 5 µg of plasmid DNA per 1*10^6^ cells per transfection and electroporated. Directly after, cells were transferred to WillCo-dishes (Cat# HBST-3522, WillCo Wells BV) with pre-warmed RPMI without antibiotics or serum. After 3 hours, the medium was replaced by RPMI supplemented with 10% (v/v) FCS and antibiotics (AA). Prior to live-cell imaging, cells were washed with PBS and imaging was performed in RPMI without Phenol red to avoid autofluorescence. Confocal imaging of transiently transfected cells was performed at 37 °C on an Olympus confocal microscope with a 63×/1.3 NA PL APO oil immersion objective (butanol experiments), or a Leica SP8 confocal microscope (PLD1-inh, PLD2-inh experiments), where the samples were excited with a 488-nm (GFP) argon and a 543-nm (RFP) NeHe laser. For TIRF microscopy cells were imaged at 37 °C on an Olympus IX-71 wide field fluorescence microscope equipped with a 3-line TIRF system (488/568/640) and a Hamamatsu ImagEM EM-CCD camera equipped with a PL APO 60×/1.4 NA oil immersion TIRF objective. During acquisition, cells were imaged untreated for a period of time varying from 10 to 20 minutes. Subsequently cells were treated with 25 mM *n*- or *t*-butanol or 5 µM PLD1-inh/PLD2-inh until complete podosome dissolution was reached. Next, cells were washed with medium. For podosome reformation experiments, fMLP was added after complete podosome dissolution at final concentration of 1 µM and imaged until complete podosome cluster reformation. For dual color imaging, images were acquired sequentially to prevent bleed through. Images were acquired every 4.6 seconds (PLD inhibitor experiments) or 6.6 seconds (butanol experiments) for confocal live cell imaging and every 1 or 2 seconds during TIRF live cell imaging.

### Image analysis

All images were analyzed using FIJI-ImageJ software version 1.52b^99^. For the percentage of podosome-forming cells, cells containing less than 10 podosomes were considered podosome negative. The percentage of podosome-forming cells was determined by manually counting the number of positive cells and the total number of cells. For each condition, five fields of view per coverslip were analyzed, leading to a total of 15 fields per condition over all three coverslips. Per condition, the number of positive counted cells and the total number of cells was averaged for all fields of view; the positive cell average was divided by the total cell number average and multiplied by 100 to obtain the percentage of cells with podosomes. For the radial intensity profile analysis of actin and PASS/Akt-PH in single podosomes (Fig. 6f,g), we first used the maxima finder of FIJI to determine the center of single podosome cores in the actin channel. Next, a line was drawn through the center and rotated 360° to calculate the radial intensity profile for each podosome in both the actin and the PASS/Akt-PH channel. This radial intensity profile was normalized for each podosome and all intensity profiles of all podosomes from a single cell were averaged. The resulting average radial intensity profiles were further averaged over multiple cells and these results were plotted with the SD. For visual representation of the actin and PASS/Akt-PH fluorescence at single podosomes (Fig. 6d,e), a 20-pixel square ROI around the center of single cores was extracted for each measured podosome. Next, the fluorescence intensity for each podosome was normalized and multiple podosomes from the same cell were averaged. Finally, a radial averaging was performed and the final image was again normalized. For the average intensity profile of reformation and dissolution of single podosomes, DCs treated for 10 minutes with 25 mM *n*-butanol and subsequently stimulated for 10 minutes with 1 µM fMLP were used for analysis. Analysis was performed during reformation of podosomes (200 second time frame before maximum actin signal) and during dissolution of podosomes (200 second timeframe after maximum actin signal). The highest actin intensity signal was chosen as reference for a fully assembled podosome. The lowest actin signal was chosen as a point of reference for a fully dissolved podosome. From that time point, we recorded the fluorescent signals of the preceding 110 frames (in total 3 minutes and 40 seconds). Arbitrarily, only podosomes that were completely formed or dissolved within 110 frames were included in the analysis. As a control, a podosome-free area in the cell was analyzed. For measuring the percentage of gelatin degradation, the fluorescence intensity underneath the cells relative to the fluorescence intensity around the cells was quantified with a semi-automated Fiji analysis macro.

### Flow cytometry

To evaluate the effect of *n*-butanol/*t*-butanol treatment or PLD1/PLD2 inhibition on integrin expression, cell surface expression of Mac-1 (α_M_β_2_ integrin), active and total β_1_ integrins, and total β_2_ integrins was assessed using flow cytometry. In short, DCs day 5 or 6 were treated in suspension with 25 mM *n*-butanol, 25 mM *t*-butanol or 5 µM PLD1-inh or PLD2-inh for 10 minutes. Subsequently, cells were spun down, washed in PBA (PBS/0.5% BSA/0.01% NaN_3_) and blocked in PBA with 2% human serum for 10 minutes at 4 °C. Subsequently cells were stained with primary antibodies in PBA for 30 minutes, washed with PBS and finally stained with secondary Goat anti-Mouse (H + L)-Alexa488 antibody for 30 minutes. Finally, cells were fixed with 4% PFA in PBS for 10 minutes and resuspended in stealth fluid. Samples were measured on a BD FACSCalibur flow cytometer (BD Biosciences). Analysis was performed with Flowjo Software (Treestar Inc) version 9.2. Cell surface marker expression is represented as MFI (mean Fluorescence Intensity) percentage over isotype control.

### RNA isolation and RT-qPCR

Total RNA from HEK293 cells and from immature DCs day 6 of 3 independent donors was isolated using Aurum Total RNA Mini Kit (Bio-Rad), according to manufacturer’s instructions. Next, cDNA synthesis was performed with 1 µg of RNA using the iScript^TM^ cDNA synthesis kit (Bio-Rad). For RT-qPCR, 3 µl of the 10-fold diluted cDNA sample was mixed in a final volume of 10 µl containing 4 µl of iQ SYBR Green Supermix (Bio-Rad) and 4 pmol of each primer. Samples were analyzed with a CFX96 Real-time System (Bio-Rad). A melting curve was obtained for each sample to ensure single product amplification. As negative controls, non-RT controls (RT-) and non-template controls (NTCs) were included. Endogenous PLD1 and PLD2 levels were normalized to GAPDH levels. Primers used: *PLD1*: 5′-GAGGTGTGGGTTTCAACAGCA-3′ and 5′-TCATTGGGAAGGCACCGGAAA-3′; *PLD2*: 5′-GAGGACACAGAGACGGAACCA-3′ and 5′-GGCCGGGTATTTGCTCCAAG-3′; *GAPDH*: 5′-CCCGCTTCGCTCTCTGCTCC-3′ and 5′-CCTTCCCCATGGTGTCTGAGCG-3′.

### Statistical analysis

Statistical analysis was performed using Prism 7 (version 7.0a). Statistical significance was tested using 2-tailed paired t-test for comparison of 2 conditions; Oneway ANOVA was performed for comparison of 3 or more conditions, using Bonferroni’s test for post-hoc analysis. For flow cytometry experiments, at least 10,000 gated cells were analyzed per sample. Differences were considered statistically significant at p < 0.05.

## Supplementary information

Supplementary Info

Supplementary Video 1

Supplementary Video 2

Supplementary Video 3

Supplementary Video 4

Supplementary Video 5

Supplementary Video 6

Supplementary Video 7

Supplementary Video 8

Supplementary Video 9
