# Supplementary material for: PLD-dependent phosphatidic acid microdomains are signaling platforms for podosome formation
Source: Sci Rep. 2019 Mar 5;9:3556. doi: 10.1038/s41598-019-39358-0 (PMC6401089; doi:10.1038/s41598-019-39358-0)
Supplement: Supplementary file 1 — Supplementary Info [file 41598_2019_39358_MOESM1_ESM.pdf]

Supplementary information

PLD-dependent phosphatidic acid microdomains are signaling platforms for podosome formation.

Matteo Bolomini-Vittori[1], Svenja FB Mennens[1], Ben Joosten[1,2], Jack Fransen[1,2], Guangwei Du[3], Koen van den Dries[1] and Alessandra Cambi[1*]

[1]Department of Cell Biology, Radboud Institute for Molecular Life Sciences, Radboud University Medical Center, Nijmegen, the Netherlands

[2]Microscopic Imaging Center, Radboud Institute for Molecular Life Sciences, Radboud University Medical Center, Nijmegen, the Netherlands

[3]Department of Integrative Biology and Pharmacology, University of Texas Health Science Center, Houston, Texas, USA.

***Corresponding author:**

E-mail: alessandra.cambi@radboudumc.nl

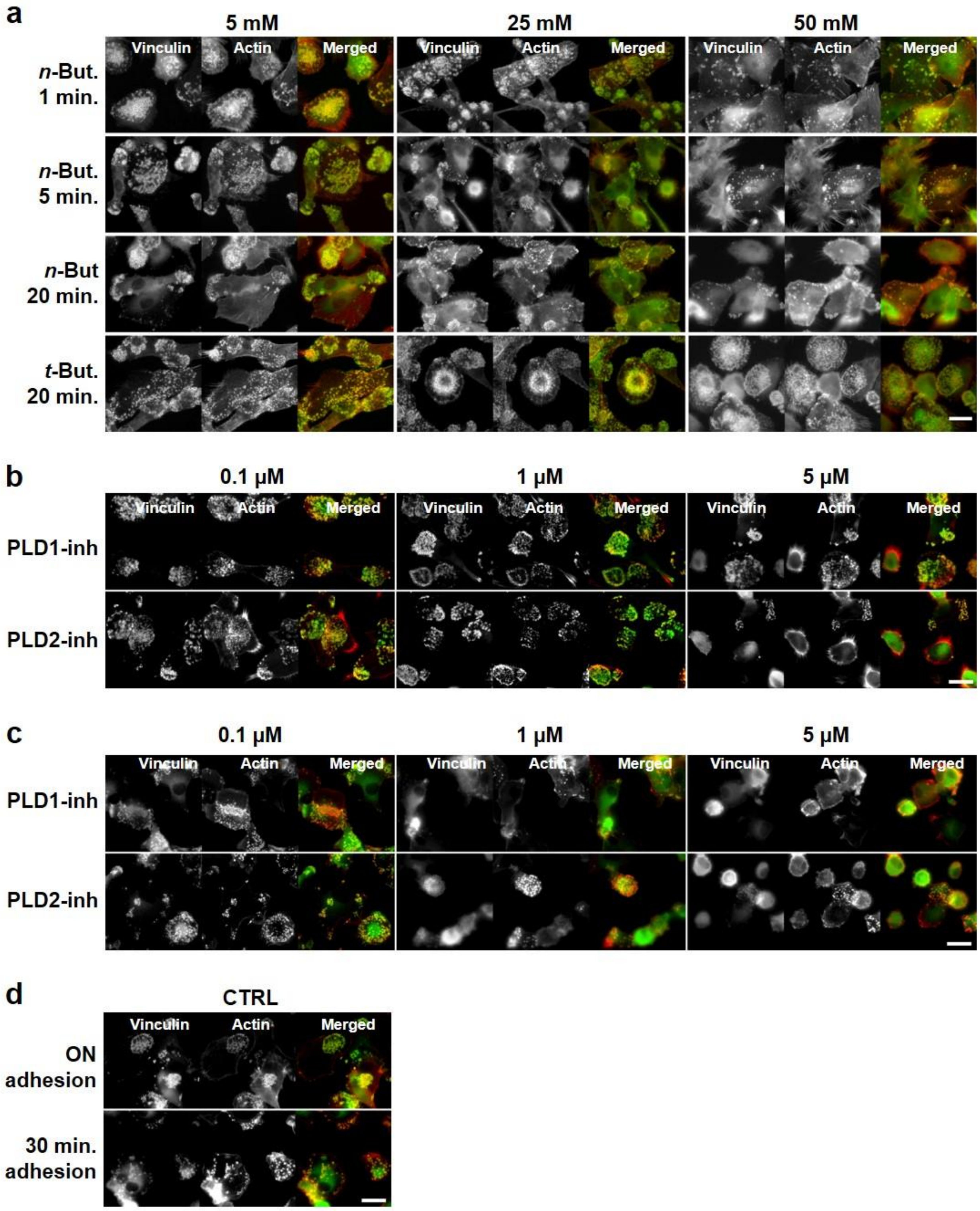
a
5 mM
25 mM
50 mM
Vinculin
Actin
Merged
n-But. 1 min.
n-But. 5 min.
n-But 20 min.
t-But. 20 min.
b
0.1 µM
1 µM
5 µM
PLD1-inh
PLD2-inh
c
0.1 µM
1 µM
5 µM
PLD1-inh
PLD2-inh
d
CTRL
ON adhesion
30 min. adhesion

Supplementary Figure S1. Detailed images of butanol-, PLD1-inh- and PLD2-inh- treated DCs.

(a) Representative widefield images of DCs treated for indicated times and at indicated doses with *n*-butanol or *t*-butanol. In the merged images vinculin is shown in green and F-actin in red. Scalebar represents 20 µm. (b) Representative widefield images of DCs adhering overnight and subsequently treated for 10 minutes and at indicated doses with PLD1-inh or PLD2-inh. In the merged image vinculin is shown in green and F-actin in red. Scalebar represents 20 µm. (c) Representative widefield images of DCs adhering for 30 minutes and subsequently treated for 10 minutes at indicated doses with PLD1-inh or PLD2-inh. (d) Representative widefield images of untreated DCs adhering overnight (upper image) or for 30 minutes (lower image). Scalebar represents 20 µm.

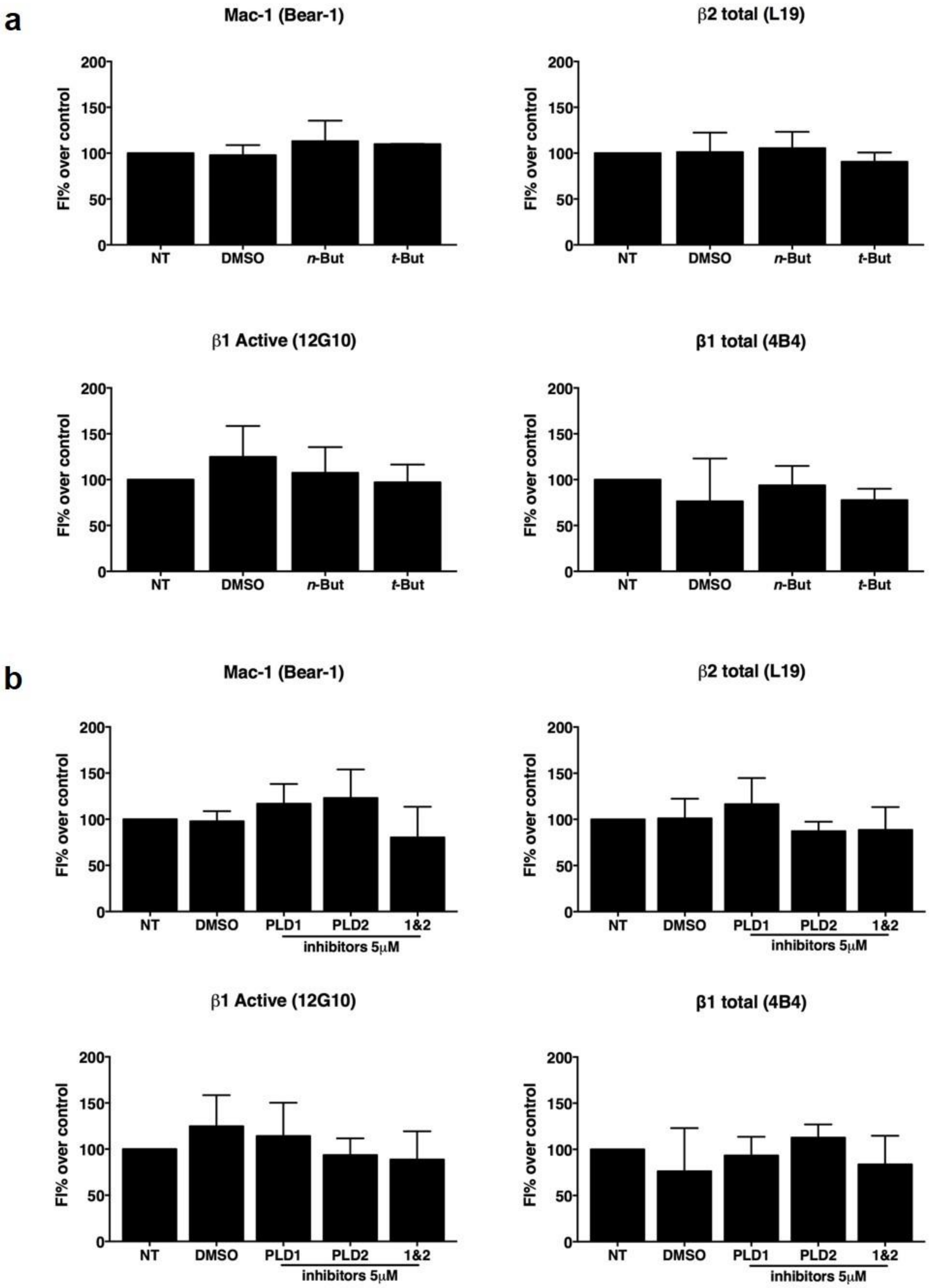

a
Mac-1 (Bear-1)
β2 total (L19)
β1 Active (12G10)
β1 total (4B4)
FI% over control
0
50
100
150
200
NT
DMSO
n-But
t-But
b
Mac-1 (Bear-1)
β2 total (L19)
β1 Active (12G10)
β1 total (4B4)
FI% over control
NT
DMSO
PLD1
PLD2
1&2
inhibitors 5μM

Supplementary Figure S2. PLD1 and PLD2 inhibition does not affect integrin surface expression.

(a) Cell surface expression of Mac-1 ($\alpha_M\beta_2$ integrin), total β_2 integrins, active β_1 integrins and total β_1 integrins in DCs treated for ten minutes with 25 mM *n*-butanol, 25 mM *t*-butanol, DMSO or non-treated. Cell surface expression is measured with flow cytometry and represented as Mean Fluorescence Intensity (MFI). Bars represent mean with SEM (3 independent experiments). (b) Cell surface expression of Mac-1 ($\alpha_M\beta_2$ integrin), total β_2 integrins, active β_1 integrins and total β_1 integrins in DCs treated for 10 minutes with 5 µM PLD1-inh or PLD2-inh (or both), with DMSO or non-treated. Cell surface expression is measured with flow cytometry and represented as Mean Fluorescence Intensity (MFI). Bars represent mean with SEM (3 independent experiments). For all the experiments statistical significance was tested with one-way ANOVA with post-hoc Bonferroni's multiple comparisons test: adjusted P-values > 0.992, which indicates that no statistically significant difference is detected in these quantifications.

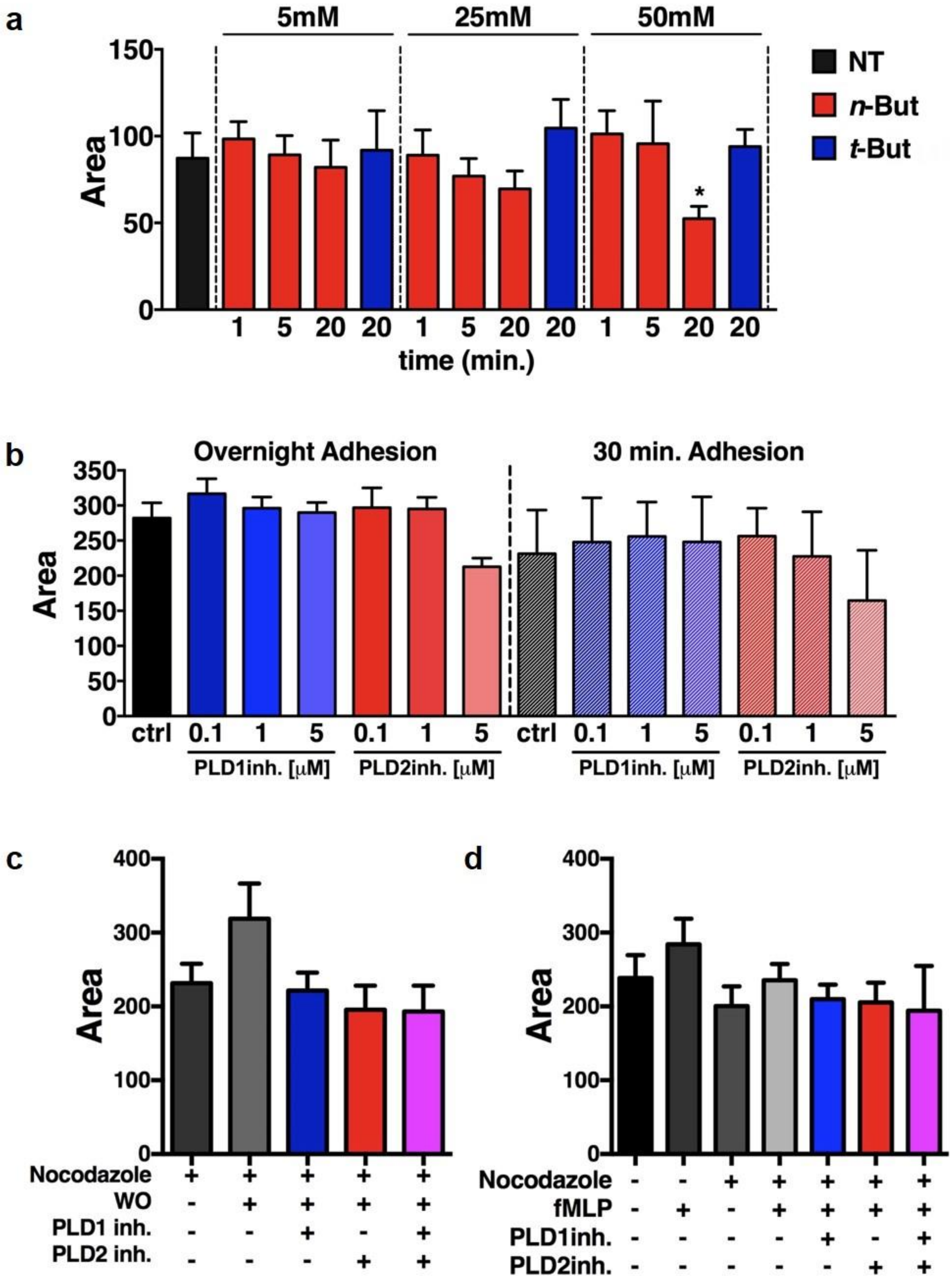
a
5mM
25mM
50mM
150
100
50
0
Area
1
5
20
20
1
5
20
20
1
5
20
20
time (min.)
*
NT
n-But
t-But
b
Overnight Adhesion
30 min. Adhesion
350
300
250
200
150
100
50
0
Area
ctrl
0.1
1
5
0.1
1
5
ctrl
0.1
1
5
0.1
1
5
PLD1inh. [μM]
PLD2inh. [μM]
PLD1inh. [μM]
PLD2inh. [μM]
c
400
300
200
100
0
Area
Nocodazole + + + + +
WO - + + + +
PLD1 inh. - - + - +
PLD2 inh. - - - + +
d
400
300
200
100
0
Area
Nocodazole - - + + + + +
fMLP - + - + + + +
PLD1inh. - - - - + - +
PLD2inh. - - - - - + +

Supplementary Figure S3. PLD1 and PLD2 inhibition does not influence cell spreading.

(a) Relative cell area of DCs treated for indicated times and at indicated doses with *n*-butanol or *t*-butanol 3 samples of each experimental condition were prepared for each experiment. A minimum of 25 cells were imaged and analyzed for each sample. 3 independent experiments were performed. Bars represent mean with SEM. (b) Relative cell area of overnight adherent and 30 minutes adherent DCs treated for 10 minutes and at indicated doses with PLD1-inh or PLD2-inh. 3 samples of each experimental condition were prepared for each experiment. A minimum of 20 cells were imaged and analyzed for each sample. 10 independent experiments were performed for overnight adhesion and 11 independent experiments for 30 minutes adhesion. Bars represent mean with SEM. (c) Relative cell area of DCs treated as follows: 20 µM nocodazole for 20 minutes. Then nocodazole was replaced (washed out; WO) by medium with or without PLD1-inh or PLD2-inh and cells were treated for an additional 10 minutes cells with or without 5 µM PLD1-inh or PLD2-inh. 3 samples of each experimental condition were prepared for each experiment. A minimum of 25 cells were imaged and analyzed for each sample. 6 independent experiments were performed. Bars represent mean with SEM. (d) Relative cell area of DCs treated as follows: 20 µM nocodazole for 20 minutes. Then nocodazole was replaced (washed out; WO) by medium with or without PLD1-inh or PLD2-inh and cells were treated for an additional 10 minutes cells with or without 5 µM PLD1-inh or PLD2-inh. Finally, fMLP was added. Cells were treated with 1 µM fMLP for 5 minutes in the presence or absence of PLD1-inh or PLD2-inh. 3 samples of each experimental condition were prepared for each experiment. A minimum of 25 cells were imaged and analyzed for each sample. 8 independent experiments were performed. Bars represent mean with SEM. For all the experiments statistical significance was tested with one-way ANOVA with post-hoc Bonferroni's multiple comparisons test: adjusted P-values > 0.9999 except for * P-value < 0.05 .

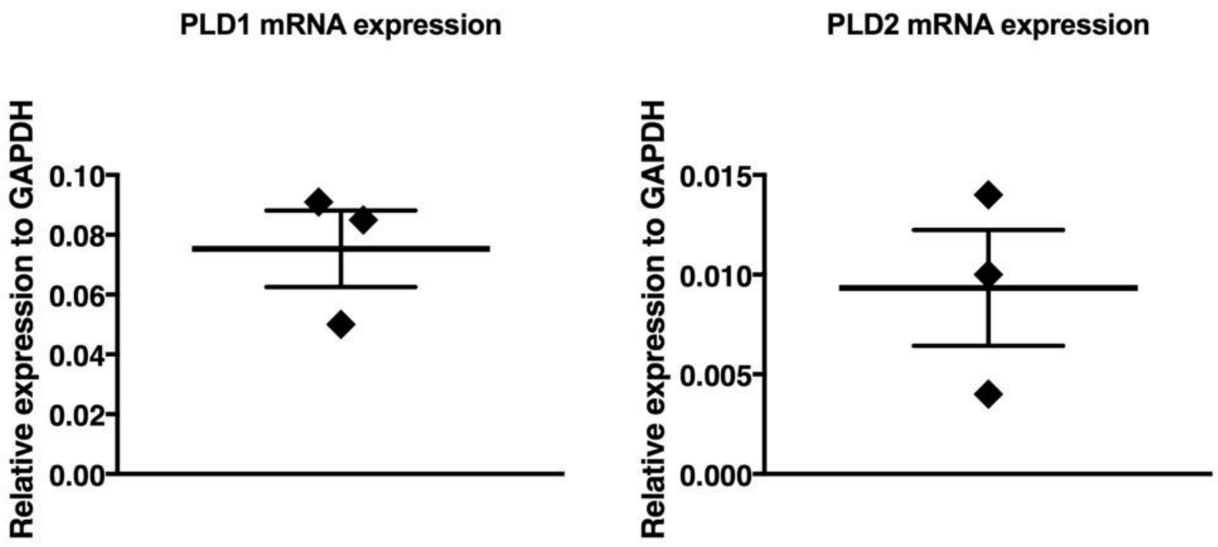

Supplementary Figure S4. Expression of PLD1 and PLD2 mRNA in DCs.

PLD1 and PLD2 mRNA expression in DCs after 6 days of differentiation. Data points represent 3 independent donors (mean of two technical replicates), with SEM. PLD1 and PLD2 mRNA expression levels are relative to GAPDH expression. PLD1 and PLD2 expression levels in HEK293 cells are indicated as dotted lines.

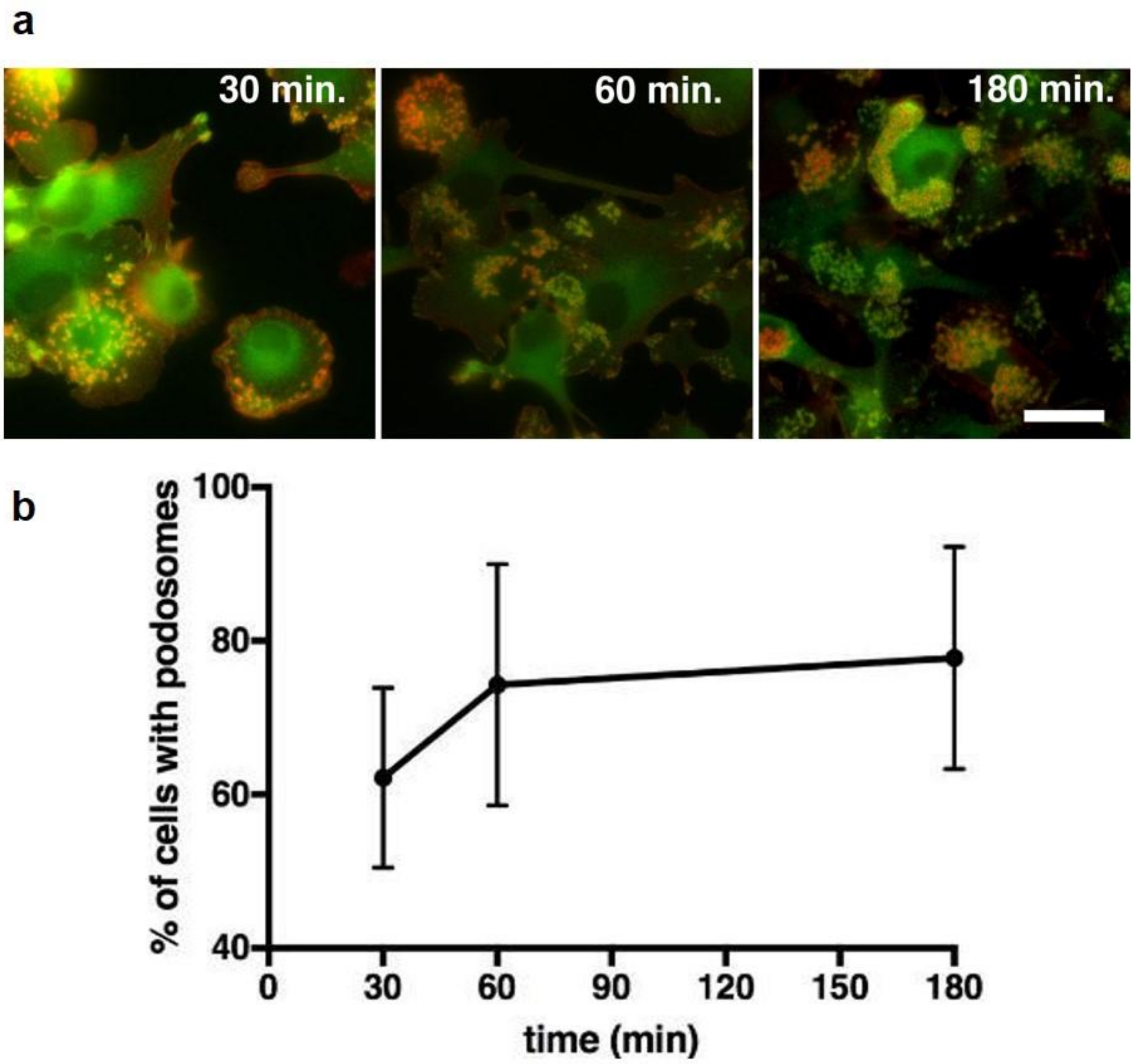

Supplementary Figure S5. Adhesion time-course.

(a) Representative widefield images of DCs seeded on fibronectin-coated glass coverslips for 30, 60 and 180 minutes. Vinculin is shown in green and F-actin in red. Scalebar represents 20 µm. (b) Percentage of cells with podosomes in DCs seeded on fibronectin-coated coverslips at indicated times. A minimum of 120 cells were analyzed per time point. 3 independent experiments were performed. Data points represent mean with SEM.

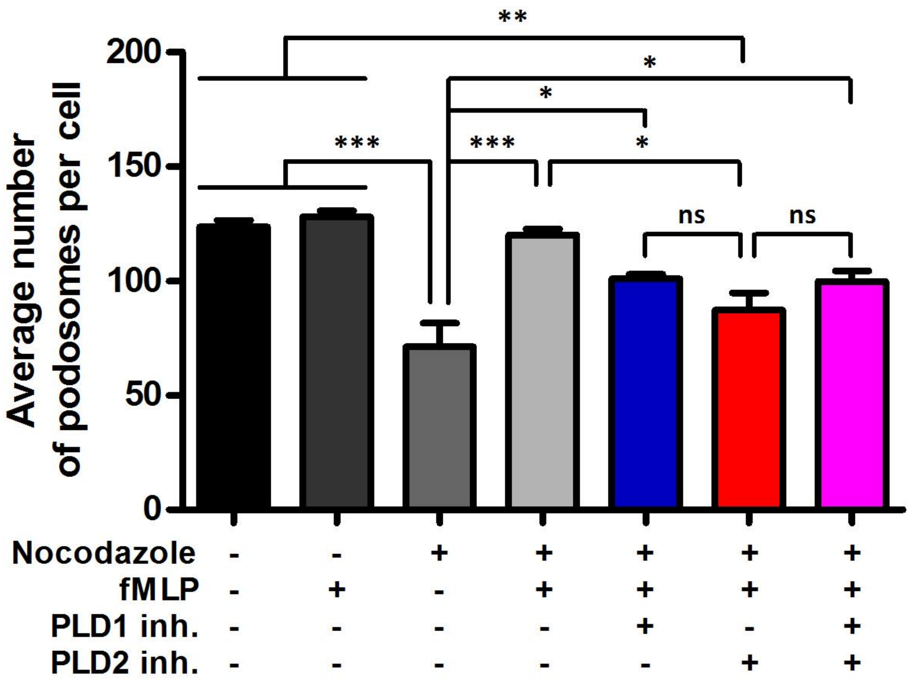

Supplementary Figure S6. PLD inhibition does not affect the average number of podosomes per cell.

Average number of podosomes per cell. DCs were treated with 5 µM nocodazole for 20 minutes. Then nocodazole was replaced (washed out; WO) by medium with or without PLD1-inh or PLD2-inh and cells were treated for an additional 10 minutes with or without 5 µM PLD1-inh or PLD2-inh. Finally, fMLP was added. Cells were treated with 1 µM fMLP for 5 minutes in the presence or absence of PLD1-inh or PLD2-inh. The number of podosomes per cell was determined by manual counting using the multi-point tool in FIJI. The Average number of podosomes per cell was determined for a minimum of 17 cells for each experimental condition. Three independent experiments were performed. Bars represent SEM. Statistical significance was tested with one-way ANOVA with post-hoc Bonferroni's multiple comparisons test. * $p < 0.05$; ** $p < 0.01$; *** $p < 0.001$

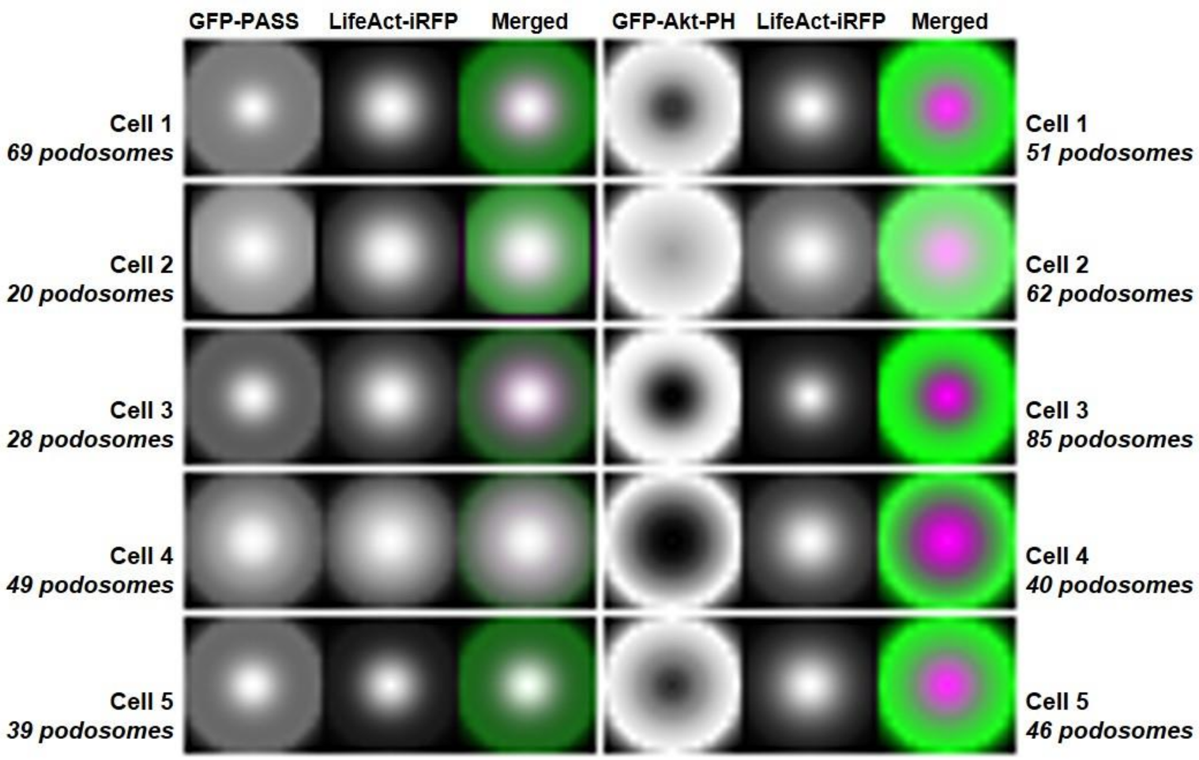

Supplementary Figure S7. 360° average intensity profile analysis confirms PA islets at podosome site. Single cell overlay of multiple individual podosomes from representative cells expressing either GFP-PASS (9 cells) or GFP-Akt-PH (5 cells), in combination with LifeAct-iRFP.

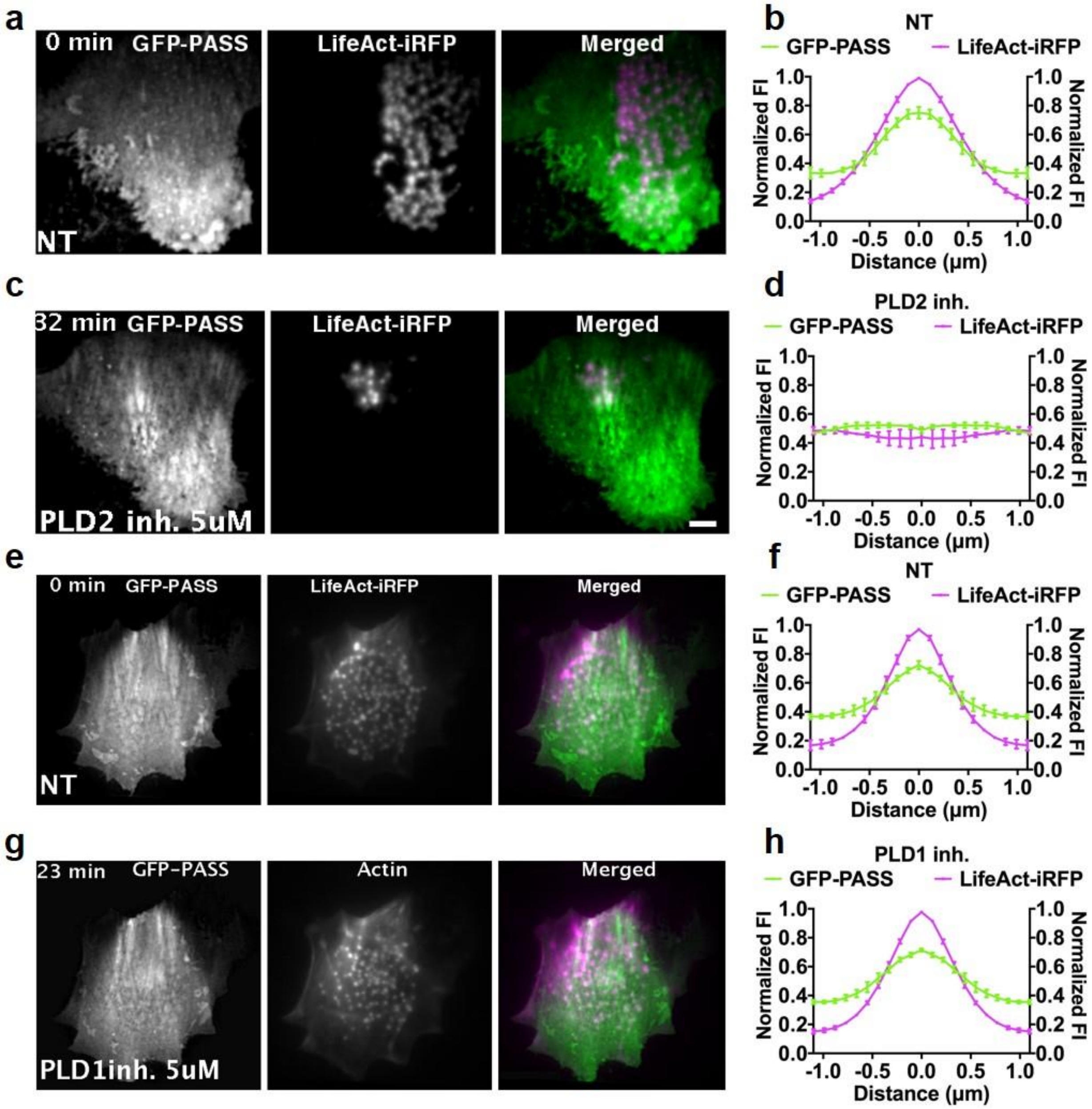

Supplementary Figure S8. PA islets are strictly connected to actin podosome presence.

(a) Representative images of TIRF live cell imaging of DCs transfected with GFP-PASS and LifeAct-iRFP, before treatment (0 min). (b) Average fluorescence intensity profile of a single podosome in DCs before treatment. (c) Representative images of TIRF live cell imaging of DCs transfected with GFP-PASS and LifeAct-iRFP after 16 minutes of treatment (32 min) with 5 µM PLD2-inh. In the merged image

LifeAct-iRFP is shown in magenta and GFP-PASS in green. Cells were imaged for 44 minutes in total at 1 frame every 1 second. Scalebar represents 5 µm. (d) Average fluorescence intensity profile of a single podosome in DCs after 16 minutes of treatment with 5 µM PLD2-inh. (e) Representative images of TIRF live cell imaging of DCs transfected with GFP-PASS and LifeAct-iRFP, before treatment with PLD1-inh (0 min). (f) Average fluorescence intensity profile of a single podosome in DCs before treatment with PLD1-inh. (g) Representative images of TIRF live cell imaging of DCs transfected with GFP-PASS and LifeAct-iRFP after 10 minutes of treatment with 5 µM PLD1-inh. In the merged image LifeAct-iRFP is shown in magenta and GFP-PASS in green. Cells were imaged for 29 minutes in total at 1 frame every 2 second. Scale bar represents 5 µm. (h) Average fluorescence intensity profile of a single podosome in DCs after 10 minutes of treatment. > 50 podosomes in total from 4 cells were analyzed for each condition. Data points/lines indicate mean with SEM. 1 pixel =110 nm

Supplementary Video 1

Live cell imaging of DCs transfected with LifeAct-GFP. After 10 minutes of imaging, DCs were treated with 25 mM *n*-Butanol. After 10 minutes of treatment, *n*-Butanol was washed out (WO). Cells were imaged for 24 minutes in total at 1 frame every 6.6 seconds.

Supplementary Video 2

Live cell imaging of DCs transfected with LifeAct-GFP. After 10 minutes of imaging, DCs were treated with 25 mM *t*-Butanol. After 10 minutes of treatment, *t*-Butanol was washed out (WO). Cells were imaged for 24 minutes in total at 1 frame every 6.6 seconds.

Supplementary Video 3

Live cell imaging of DCs transfected with LifeAct-GFP. After 6 minutes of imaging, DCs were treated with 5 µM PLD1-inh. Cells were imaged for 25 minutes in total at 1 frame every 4.6 seconds.

Supplementary Video 4

Live cell imaging of DCs transfected with LifeAct-GFP. After 6 minutes of imaging, DCs were treated with 5 µM PLD2-inh inhibitor. Cells were imaged for 25 minutes in total at 1 frame every 4.6 seconds.

Supplementary Video 5

TIRF live cell imaging of DCs transfected with GFP-PASS in combination with LifeAct-iRFP. In the merged image LifeAct-iRFP is shown in magenta and GFP-PASS is shown in green. Cells were imaged for 23 minutes in total at 1 frame every 0.5 seconds.

Supplementary Video 6

TIRF live cell imaging of DCs transfected with GFP-Akt-PH, in combination with LifeAct-iRFP. In the merged image LifeAct-iRFP is shown in magenta and GFP-Akt-PH is shown in green. Cells were imaged for 14 minutes in total at 1 frame every second.

Supplementary Video 7

TIRF live cell imaging of DCs transfected with GFP-PASS and LifeAct-iRFP treated with 5 μM PLD2-inh. In the merged image LifeAct-iRFP is shown in magenta and GFP-PASS in green. Cells were imaged for 44 minutes in total at 2 frames every second.

Supplementary Video 8

TIRF live cell imaging of DCs transfected with GFP-PASS and LifeAct-iRFP treated with 5 μM PLD1-inh. In the merged image LifeAct-iRFP is shown in magenta and GFP-PASS in green. Cells were imaged for 23 minutes in total at 2 frames every second.

Supplementary Video 9

TIRF live cell imaging of DCs transfected with GFP-PASS and LifeAct-iRFP, before treatment (NT), subsequently treated for 10 minutes with 25mM *n*-Butanol and finally stimulated with 1 μM fMLP. In the merged image LifeAct-iRFP is shown in magenta and GFP-PASS is shown in green. Cells were imaged for 36 minutes in total at 1 frame every second.
